# Synergistic effects of terpolymer and its oleic modified nano-bentonite nanocomposite for cold flow enhancement for diesel fuel

**DOI:** 10.1038/s41598-025-24090-9

**Published:** 2025-11-10

**Authors:** Abeer A. El-Segaey, Asmaa A. Roshdy, Taisir T. Khidr, Hoda A. Mohammed

**Affiliations:** 1 Petroleum Applications Department, Egyptian Petroleum Research Institute, Nasr City, Cairo, Egypt; 2 Petrochemicals Department, Egyptian Petroleum Research Institute, Nasr City, Cairo, Egypt; 3 Analysis and Evaluation Department, Egyptian Petroleum Research Institute, Nasr City, Cairo, Egypt

**Keywords:** Terpolymer, Oleic modified nano-bentonite, Pour point depressant, Polarized optical microscope, Flow improver, Diesel fuel, Chemistry, Engineering, Materials science, Nanoscience and technology

## Abstract

The defect in the properties of diesel fuel at low temperatures, caused by wax crystallization and agglomeration, negatively impacts engine performance at these temperatures. This work synthesizes and evaluates modified new terpolymeric additives, hexadecylacrylate-octyoleate-hexadecene (TPO), and its nanonentonite (NTPO), as depressants for the pour point of the diesel fuel (PPDs) and cold flow improvers. These polymers were synthesized using the prepared esters of hexadecylacrylate and octyoleate with 1-hexadecene by adding 1% modified nano-bentonite with oleic acid (NB-OL) for NTPO. The additives were characterized using FT-IR, ^1^H NMR, GPC, TEM, EDX, and DLS to analyze their structural and morphological properties. The thermal degradation was also studied through TGA and DSC to prove the synergistic effect of the NTPO, while a polarized optical microscope was used to analyze wax crystal shape to show tiny and dispersant wax at NTPO. Diesel fuel treated with TPO and NTPO was tested for pour point, viscosity, and cold flow behavior using ASTM standard methods. Results demonstrated that TPO reduced the pour point by up to 24 °C, while NTPO achieved a 30 °C reduction, significantly improving low-temperature flow performance compared to conventional PPDs. The Viscosity index was increased from 116 to 120.3 and 126.85 for both TPO and NTPO. The enhanced efficiency of NTPO was attributed to the synergistic effect between the polymer matrix and nanobentonite (NB-OL), which effectively inhibited wax crystal growth and modified crystal morphology. This study presents a promising approach for developing high-performance, cost-effective additives to enhance diesel fuel operability in cold environments.

**Supplementary Information:**

The online version contains supplementary material available at 10.1038/s41598-025-24090-9.

## Introduction

Diesel fuel, a petroleum-based product, primarily consists of hydrocarbon chains ranging from C_8_ to C_30_^1^. These hydrocarbons significantly impact fuel transportation and engine performance^2–5^. At lower temperatures, their solubility drops, causing wax separation due to Van der Waals forces between hydrocarbon chains, which leads to wax crystal formation^6–8^. As temperatures continue to fall, these paraffin crystals expand, forming a network that traps liquid fuel and disrupts cold flow properties^9–11^. The most effective approach to address this issue involves incorporating pour point depressants (PPDs) into diesel fuel to lower its pour point by^12–16^. These additives typically consist of high-molecular-weight polymers featuring numerous polar functional groups, including hydroxyl and carboxyl moieties^17–19^. Through Van der Waals forces and hydrophobic interactions, these polymers associate with hydrocarbon molecules in the oil, forming structured complexes where the hydrophilic components orient outward, effectively inhibiting wax crystal agglomeration and precipitation. The mechanism of PPD action is well-explained by three established theories: crystal nucleation, adsorption, and eutectic formation. These theoretical frameworks not only elucidate how PPDs enhance diesel fuel flowability but also provide crucial insights for developing more efficient pour point depressant formulations^20–22^.

Pour point depressants (PPDs) provide an economical approach for improving the low-temperature fluidity of oils by altering wax crystallization behavior. These additives feature a dual molecular structure: a paraffin-like segment that interacts with wax crystals and a polar component that alters crystal morphology^23–27^. Effective PPD design requires specific structural features, including an optimal number of long alkyl side chains, proper spacing between hydrocarbon groups, and balanced monomer ratios in copolymer formulations^28–31^. While PPDs significantly improve low-temperature flow properties by slowing wax precipitation and disrupting crystal growth, they do not reduce the overall quantity of precipitated wax crystals^32–34^. PPDs that are available in the market comprise polymethacrylates (PMA)^34–37^, ethylene–vinyl acetate copolymers (EVA)^38,39^, maleic anhydride copolymers^40,41^, and poly-α-olefin (PAO) derivatives^42^. The effectiveness of these additives depends on their molecular architecture, where a balanced combination of polar and non-polar functional groups works synergistically to improve diesel’s cold flow properties. These structural components collectively lower both the cold filter plugging point (CFPP) and pour point (PP) by modifying wax crystallization behavior, thereby maintaining fuel flowability at reduced temperatures^43^.

Conventional polymer-based pour point depressants (PPDs) face challenges such as fuel-specific selectivity and reduced performance under thermal/ shearing stress. To address these limitations, researchers have recently explored polymer-inorganic nanocomposites, inspired by their proven ability to enhance mechanical, thermal, and functional properties, like electrical, magnetic, and optical properties, in other industrial applications^44–47^. By incorporating nanoparticles like silica^38^, clay^48^, graphene oxide^49^, carbon nanotubes^50^, or magnetic Fe_3_O_4_^51^ into PPD matrices, these hybrid additives create nucleation sites that effectively modify wax crystal morphology and improve oil rheology^52,53^. The nanocomposite PPDs demonstrate superior performance in inhibiting wax deposition and maintaining flow-ability compared to traditional polymers. However, achieving homogeneous dispersion of inorganic nanoparticles within organic polymer matrices remains challenging, often requiring surface modification of nanoparticles prior to incorporation, a process that adds complexity to PPD formulation^54,55^.

Incorporating a terpolymer nanocomposite enhances the properties as absorption ability, charge mobility, and solubility. Terpolymer introduces different function groups by using functional monomers^56^; methacrylate, oleic acid and α-olefins. By introducing functional monomers into its molecular structure, the structure of hexadecyl acrylate can be well adjusted^57,58^. Oleic acid increases the polarity^6^ and α- olefins increase the molecular weight of the terpolymer leading to enhance the properties of the oil^59^.

This study synthesized a new terpolymer-based flow improver (TPO) and its nanocompsit (NTPO) using low cost commercial materials to ease its applicability; oleic acid, methacrylic acid and α-olefins. The polymerization operated with hexadecylacrylate, octyoleate ester, and 1-hexadecene via free radical solution polymerization, along with its modified nano-bentonite with oleic acid composite. TPO and NTPO were use as PPDs in diesel fuel. The synthesized additives underwent purification and characterization using FT-IR spectroscopy, ^1^HNMR, thermal analysis (TGA and DSC), GPC and dynamic light scattering (DLS) to analyze their chemical structure and particle size distribution. Polarized optical microscopy (POM) analysis revealed modified wax crystal morphology in treated diesel fuel. The synthesized additives were tested for their effectiveness as pour point depressants and for improving the rheological properties of diesel fuel.

## Experimental section

### Materials and chemicals

**Fig. 1:**
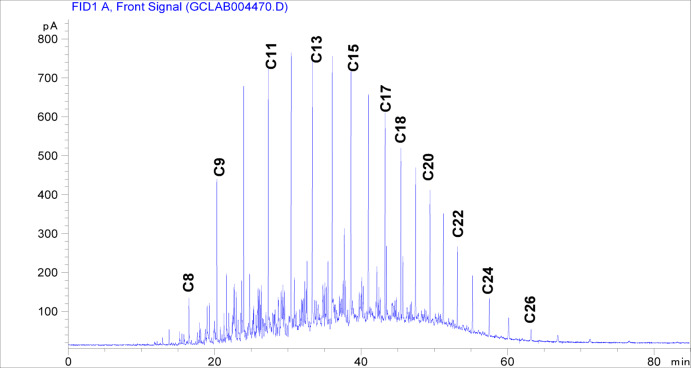
Carbon number distribution of the tested diesel fuel obtained from gas chromatography (GC) analysis.

Oleic acid (C_18_H_34_O_2_, 98%), methacrylic acid (C_4_H_6_O_2_, 97%), 1-octanol (C_8_H_17_O, 98%), hexadecyl alcohol (C_16_H_34_O, 98%), nano-bentonite (BT), hydroquinone (HQ), *p*-toluene sulfonic acid (*p*-TSA), benzoyl peroxide (BPO) and anhydrous sodium hydroxide (NaOH) were procured from Sigma-Aldrich company. Solvents (xylene, ethanol, n-heptane and toluene) were acquired from Adwic Company, while the diesel fuel samples used in this study were sourced by Co-operation Company (Egypt), with their physicochemical properties comprehensively listed in Table 1 and carbon number distribution of the tested diesel fuel showing a predominant range of C_10_–C_22_ in Fig. 1.

**Table 1:** The properties of the used diesel fuel.

Property	Method	Result
Denisty at 20 °C (kg/m^3^)	SH/T0604	820.1
Denisty at 15 °C (kg/m^3^)	SH/T0604	833.1
Kinamatic viscosity at 40 °C (mm^2^/sec)	ASTM D-445	3.35
Kinamatic viscosity at 100 °C (mm^2^/sec)	ASTM D-445	1.308
Flash point (°C)	ASTM D-93	55
Pour point, (°C)	ASTM D-96	− 3
Average Carbon Number (n)	IP 372/85 (GLC)	18.3
Cetane number	ASTM D-613	51
Sulfur content (% mass)	ASTM D-4	0.36821
Boiling distillate (^o^C)	GB/T258	283–379
Acid value (mg KOH/g oil)	GB/T258	1.96

### Synthesis of esters and polymers

#### Synthesis of ester HDM (hexadecyl methylacrylate)

**Scheme 1:**
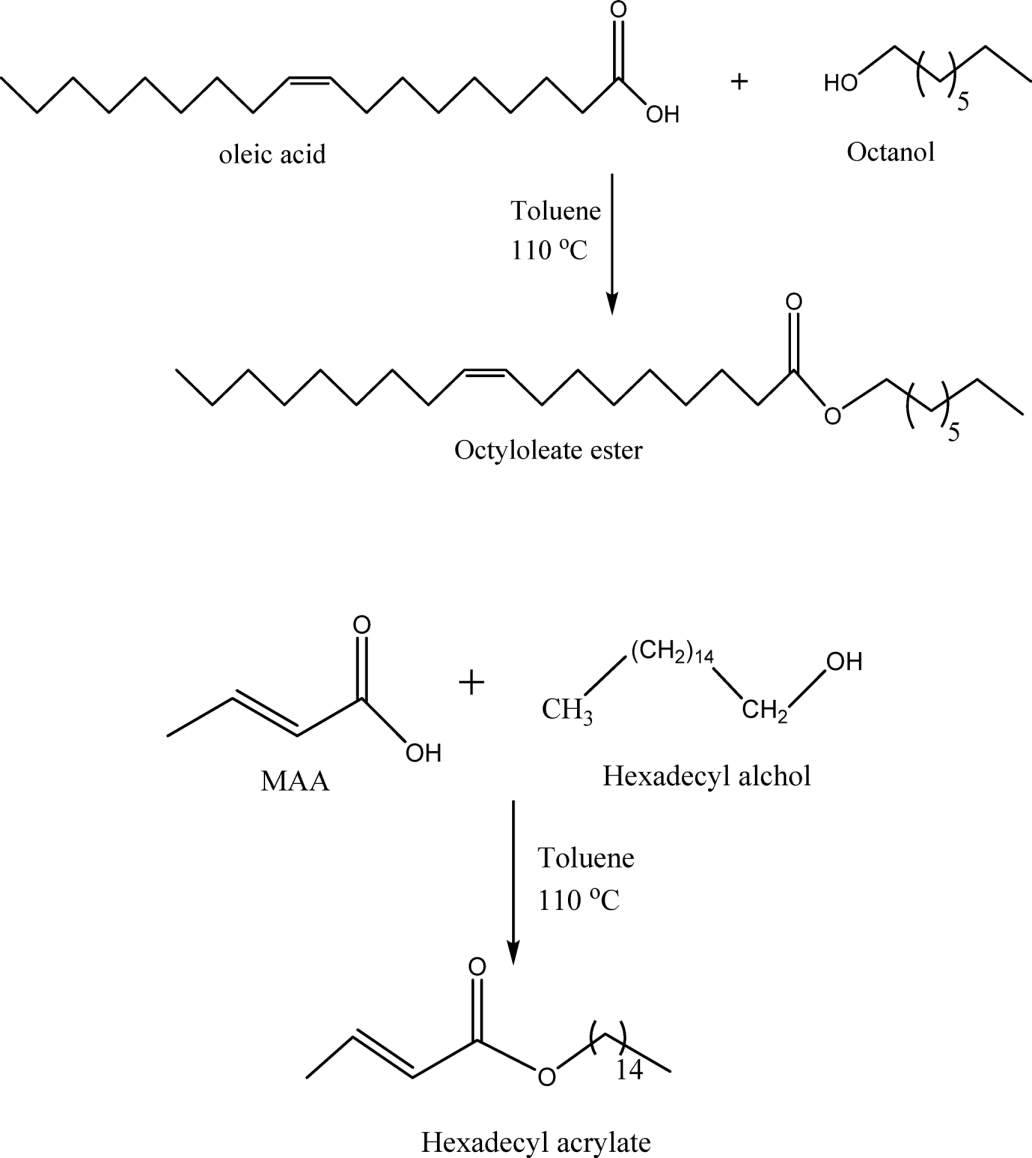
Synthesis of hexadecylacrylate and octyloleate esters.

The esterification process of methacrylic acid with cetyl alcohol was carried out in toluene, in the presence of *p*-TSA, HQ, and N_2_ inlet using the closed system with a Dean-Stark to trap the water^60^. Cetyl alcohol with hydroquinone in toluene at 60 °C for 0.5 h was sonnicated, followed by the addition of methacrylic acid and PTSA, and the temperature was elevated to 110 °C for 8 h until complete water separation. Then purification with 5% sodium hydroxide solution until neutralization, followed by distilled water rinses and rotary evaporation to collect the product. The scheme of reaction is clear in Scheme 1.

#### Synthesis of ester OE (octyloleate)

Octyloleate ester (OE) was synthesized with the mentioned method in presence of oleic acid (0.1 mol), octanol (0.1 mol) toluene (50 mL), catalyzed by *p*-toluenesulfonic acid (PTSA) and hydroquinone (HQ) as a polymerization inhibitor. The reaction followed the same procedure as before (Scheme 1), yielding 80% pure product.

#### Synthesis of the terpolymer (TPO)

The terpolymer was synthesized via free-radical solution polymerization by reacting equimolar amounts (0.1 mol each) of hexadecyl methacrylate (HDM), octyloleate ester (OE), and 1-hexadecene in xylene, using benzoyl peroxide initiator (1%) and N_2_ flow. The temperature was gradually increased to 120 °C and kept under reflux for 8 h. The product was isolated by precipitation in excess ethanol then vacuum oven.

#### Modification of nano-bentonite with oleic acid (NB-OL)

The weighted amount (0.5 g) of nano-bentonite was sonicated in 250 mL of n-heptane for half an hour; then oleic acid (4 mL) was added under the sonication for another half hour with a nitrogen flow, and the mixture for 8 h. The modified nano-bentonite with oleic acid (NB-OL) was collected by centrifuge and purified by using heptane to obtain NB-OL^61^.

#### Synthesis of the terpolymer nanocomposite (NTPO)

**Scheme 2:**
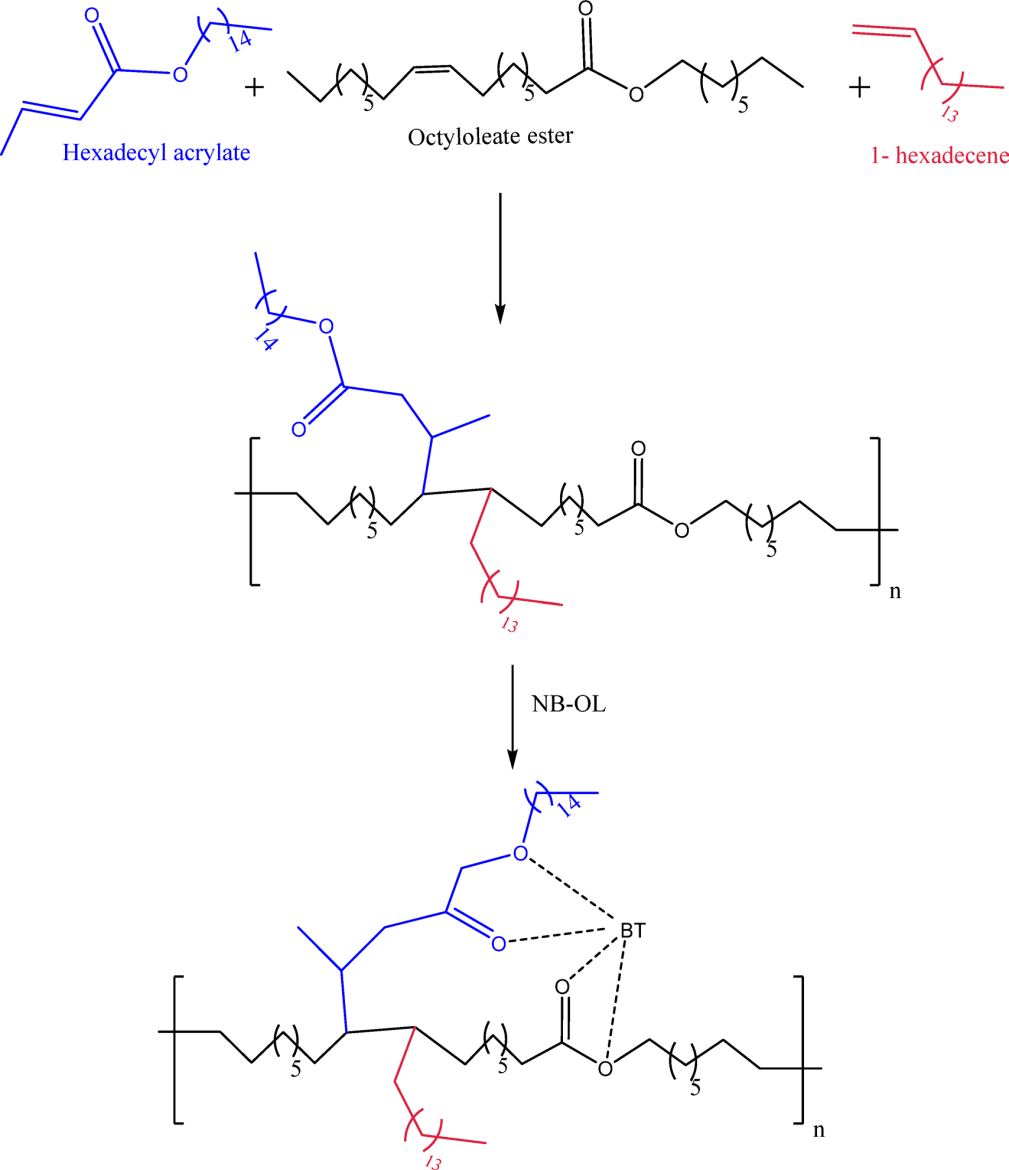
Synthesis of terpolymer (TPO) and its nanocomposite (NTPO).

To ensure that the nanoparticles are evenly distributed throughout the polymer, NB-OL 1 wt% was first dispersed in toluene and ultrasonicated for 10 min. After that, they were combined with TPO polymer and agitated for an additional 10 min. Subsequently, the mixture was transferred to a vacuum oven and dried at 100 °C for 48 h. The scheme of reaction is clear in Scheme 2.

### Investigation methods

The chemical structures of TPO and NTPO were examined using a Thermo-Fisher Nicolet iSTM10 FTIR spectrometer (USA) with a scanning range of 400–4000 cm^−1^ and resolution of 0.5 cm^−1^. Samples were prepared as KBr pellets by homogenizing the polymer with potassium bromide and compressing it into thin wafers.

Nuclear magnetic resonance spectra (^1^H NMR) were acquired on a Bruker 300 MHz spectrometer using dimethyl sulfoxide (DMSO) as solvent (10 wt% concentration). Chemical shifts were referenced against tetramethylsilane (TMS) as an internal standard to verify molecular structures of reactants and products.

Thermogravimetric analysis (TGA) and differential scanning calorimetry (DSC) were employed to evaluate the thermal stability of the synthesized terpolymers (TPs) and their nanocomposite (NTPs). The measurements were carried out using a SHIMADZU DTG-60 thermal analyzer equipped with an alumina crucible under an air atmosphere, at a constant heating rate of 10 °C/min, over a temperature range from room temperature up to 550 °C.

Gel permeation chromatography (GPC, HLC-8320) was utilized to determine the molecular weight distribution and polydispersity of the prepared sample TPO.

High-resolution transmission electron microscopy (HRTEM, JEOL JEM-2100 LaB6, Japan) at 200 kV acceleration voltage visualized the NTPO nanostructure. Samples were prepared by depositing ethanol-dispersed nanobentonite (15 min sonication) onto TEM grids.

The elemental composition of the nanocomposites was verified using energy-dispersive X-ray spectroscopy (EDX, Oxford Instruments X-Max, UK) operating at 30 kV, which was coupled with the high-resolution transmission electron microscope (HR-TEM) for simultaneous imaging and chemical analysis.

Dynamic light scattering (DLS, Brookhaven + 90) measured nanoparticle size distribution.

### Evaluation

*Evaluation of polymer performance as pour point depressants* The pour point depression efficiency of the synthesized additives was evaluated according to ASTM D97 methodology; the oil was injected with various concentrations (1000, 3000, 5000, 10,000, and 15,000 mg/L) of TPO and NTPO. The different concentration was poured into the standard jars to measure the pour point temperature (PPT). PPT was determined by cooling samples in 3 °C increments until flow cessation occurred, recording the last flowing temperature as the pour point temperature (PPT). The test was repeated three times to ensure the accuracy of the results.

*The cloud point (CP)* of diesel fuel and additive-containing samples was determined according to ASTM D2500. The test sample was cooled in a controlled cooling bath at a rate of approximately 1.5 ± 0.5 °C/min. The temperature at which the first visible cloudiness due to wax crystal formation appeared was recorded as the cloud point. All measurements were performed in duplicate to ensure repeatability, with results reported in °C.

*Viscosity-index assessment* The viscosity index (VI) enhancement was quantified using ASTM D2270 protocols using an abhold glass capillary viscometer (calibrated with certified viscosity standards, Cannon Instruments, USA) and placed in a constant-temperature bath (± 1 °C accuracy). The optimum concentration (10,000 mg/L) of additives was measured. Kinematic viscosity measurements at 40 and 100 °C for both treated and untreated diesel samples were conducted in triplicate to ensure reproducibility, demonstrating the additives’ efficiency as viscosity improvers.

*Rheological behavior* The flow behavior of diesel fuel, both with and without 10,000 mg/L TPO/NTPO additives, was evaluated as a function of temperature using an Anton Paar MCR302 rheometer equipped with precise temperature regulation. Tests conducted at 20 °C, 30 °C, and 40 °C under constant shear rate (5 s^−1^) quantified how the additives modified cold-flow behavior. Each experiment was repeated three times, and the results are reported as mean ± standard deviation.

*Wax appearance temperature (WAT)* The wax appearance temperature (WAT) of the diesel samples was determined by monitoring the change in kinematic viscosity with decreasing temperature. The viscosity was measured at different temperatures using a standard viscometer (ASTM D445). The obtained viscosity–temperature data were plotted, and the WAT was identified as the temperature corresponding to the deviation point in the linear trend of the viscosity curve, which indicates the onset of wax crystal formation.

*Ash content* The ash content of the fuel samples was determined according to ASTM D482. A known mass of each sample was placed in a pre-weighed crucible and gradually heated to remove volatile matter, followed by combustion in a muffle furnace at 775 ± 25 °C until all organic components were fully oxidized. The crucible was cooled in a desiccator and reweighed to obtain the mass of the residual ash. The ash content was then calculated as the percentage of the original sample mass. This method indicates the non-combustible inorganic matter present in the fuels. The analysis was performed before and after the addition of the prepared additives (TPO and NTPO).

*Wax crystal morphology* in diesel fuels was examined at 0 °C using a Leica DM2500P polarized optical microscope (200 × magnification, Germany), with a scale bar of 100 µm for both diesel fuel and that treated with TPO, but a scale bar of 50 µm was used for that treated with NTPO. Crystal sizes were measured using ImageJ for at least 100 crystals per sample. The average crystal size (µm) and standard deviation were calculated to quantify size distribution. Samples with TPO and NTPO additives showed smaller average crystal sizes and reduced standard deviation, indicating effective inhibition of wax crystal growth and aggregation.

## Results and discussion

### Affirmation of the structures of the prepared polymers

**Fig. 2:**
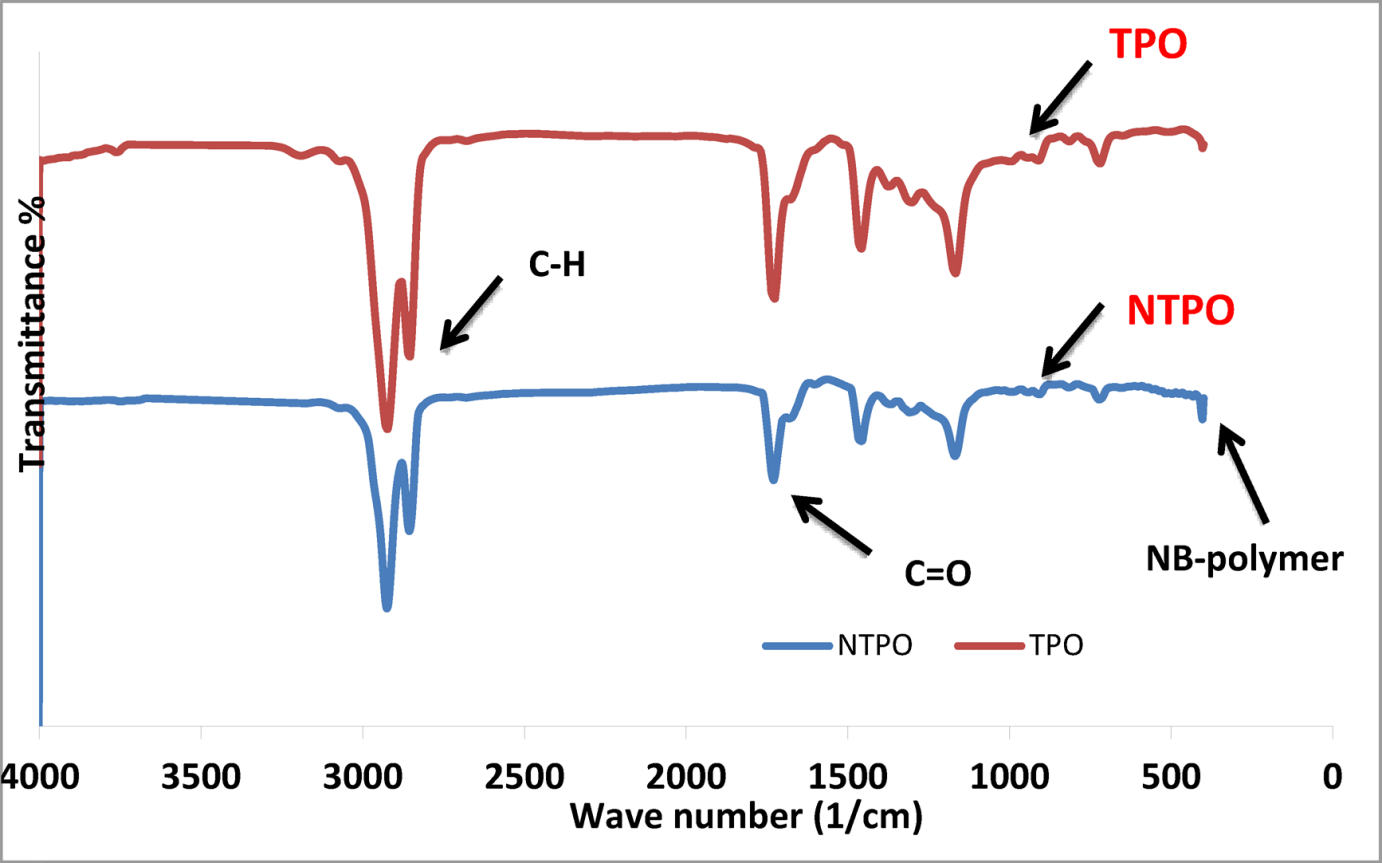
FT-IR spectrum of terpolymer (TPO) and terpolymer nanobentonite.

*IR* FT-IR spectra of the prepared polymers; TPO and NTPO were illustrated in Fig. 2. The peaks were analysed in Table 2, where the peaks of esters, C=O at 1730–1750 cm^−1^, C–H stretches at 2800–2900 cm^−1^, C–H bending at 1160–1169 cm^−1^ and presence a peak at 465 cm^−1^ at refers to the NB-OL bond with the polymer.

**Table 2:** FTIR and ^1^H NMR assignments of TPO and NTPO.

Technique	Peak position/δ (ppm)	Assignment
FTIR	1730–1750 cm^−1^	C=O stretching (ester)
FTIR	2800–2900 cm^−1^	C–H stretching
FTIR	1160–1169 cm^−1^	C–H bending
FTIR (NTPO)	465 cm^−1^	NB–OL bond (nano-bentonite)
^1^H NMR	0.8–0.9 ppm	–CH_3_ protons
^1^H NMR	1.4 ppm	–CH_2_–CH_3_ protons
^1^H NMR	2.3–2.5 ppm	–CH_2_–C = O
^1^H NMR	3.5–3.9 ppm	–CH_2_–O–

^*1*^*H NMR* The ^1^H NMR spectra of the synthesized polymers; TPO and NTPO (Fig. 3a, b) exhibited characteristic proton signals (δ, ppm) at 0.8–0.9 ppm (–CH_3_), 1.4 ppm (–CH_2_–CH_3_), 2.3–2.5 ppm (–CH_2_– C=O), and 3.5–3.9 ppm (–CH_2_–O–), confirming the expected polymer structure and successful Synthesis. As mentioned in Table 2.

**Fig. 3:**
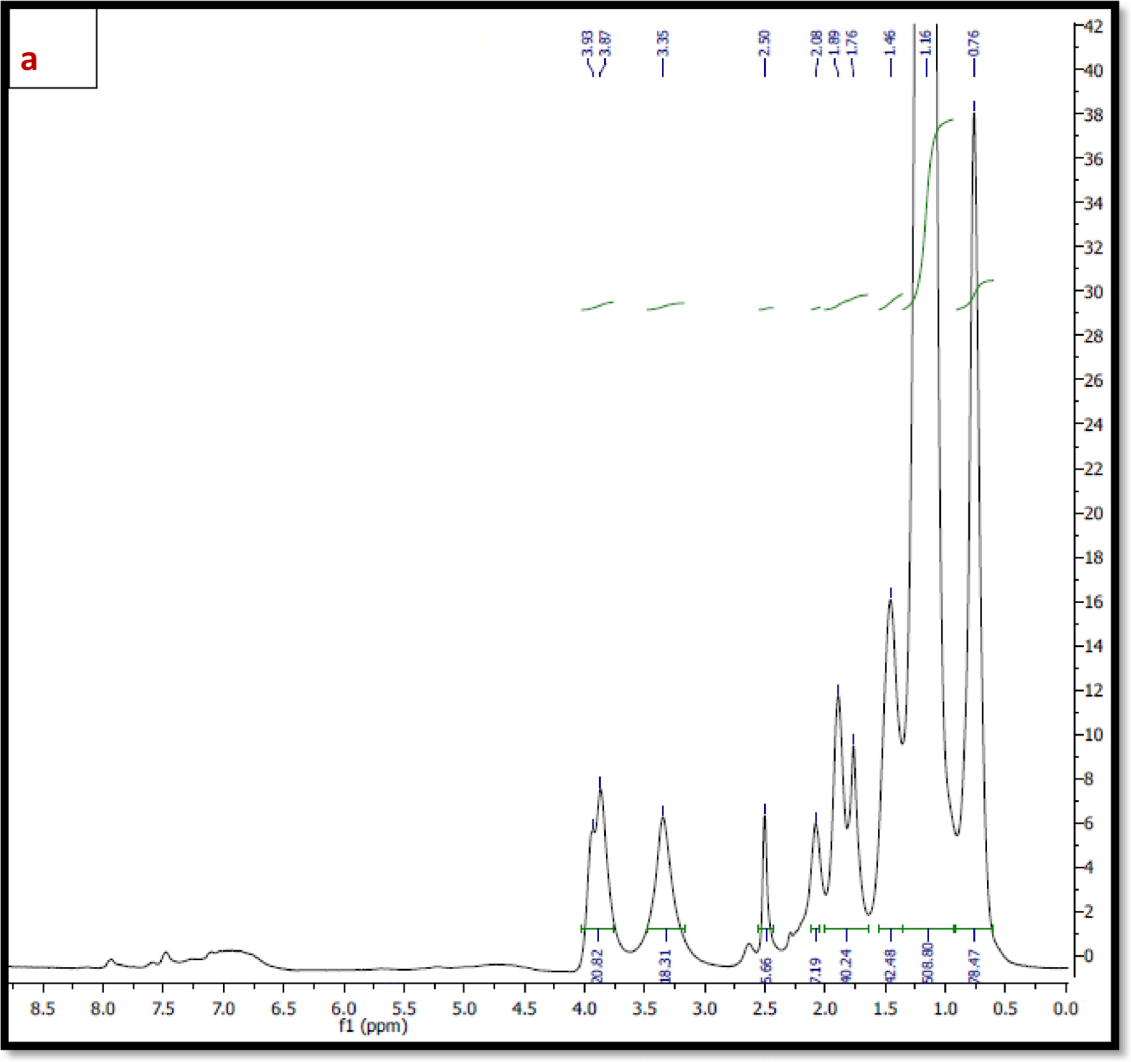

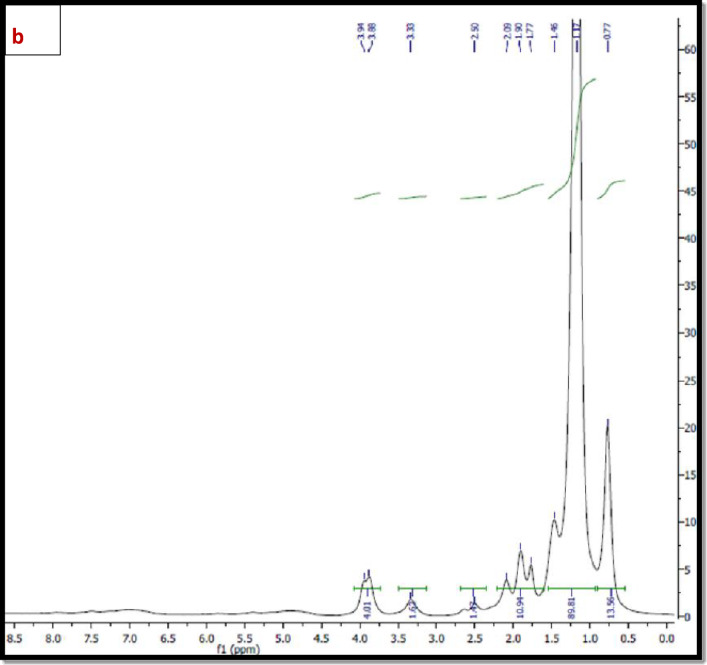
(**a**) ^1^H NMR spectrum of Terpolymer (TPO), (**b**) ^1^H NMR spectrum of nano-bentonite polymer NTPO.

**Fig. 4:**
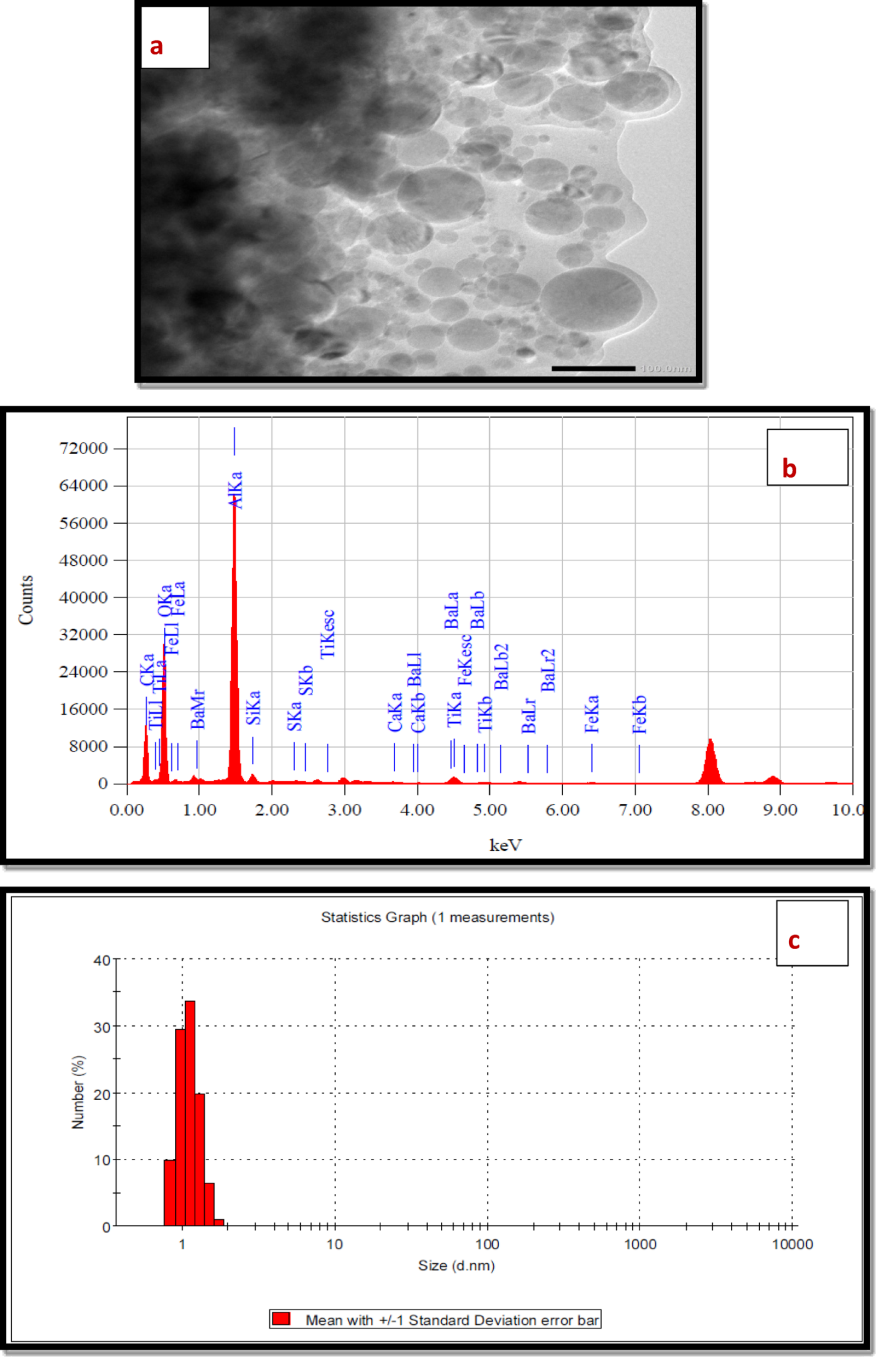
(**a**) TEM, (**b**) EDX and (**c**) DLS of the prepared nano-bentonite polymer (NTPO).

*HRTEM* The HRTEM analysis image of the polymer nanocomposite (NTPO) is shown in Fig. 4a. The TEM image clearly shows the distribution of nano-bentonite particles within the polymer matrix, where the large dark regions represent the bulk polymer phase and the bright white dots represent the embedded nano-bentonite. This distribution throughout the polymer confirms the successful Synthesis of the NTPO nanocomposite.

*EDX spectra* The EDX analysis of the nanocomposite showed intense carbon and oxygen peaks from the polymer matrix, along with distinct signals for silicon, aluminum, magnesium, potassium, and calcium – elements characteristic of nano-bentonite. These findings confirm the successful incorporation and dispersion of the nano-bentonite particles within the terpolymer structure, as evidenced in Fig. 4b. The quantitative analyses of EDX were mentioned in Table 3.

**Table 3:** EDX elemental composition of NTPO.

Element	Detected signal	Source
C, O	Strong peaks	Polymer backbone
Si, Al	Clear peaks	Nano-bentonite
Mg, K, Ca	Minor peaks	Natural bentonite impurities

*DLS* The DLS analysis of the NTPO nanocomposite sample revealed a narrow size distribution with particles below 10 nm, as visible in Fig. 4c. The measurement scale clearly shows particles around 2 nm, confirming the successful formation of the nanohybrid material. These results demonstrate the effective production of the polymer nanocomposite with well-controlled nanoparticle dimensions. The analysis of the Figure was discussed in Table 4.

**Table 4:** DLS particle size distribution of NTPO nanocomposite.

Parameter	Value (approx.)
Mean particle size	~ 2 nm
Standard deviation (SD)	Narrow (low variation)
Polydispersity Index (PDI)	< 0.2
Distribution range	< 10 nm

**Fig. 5:**
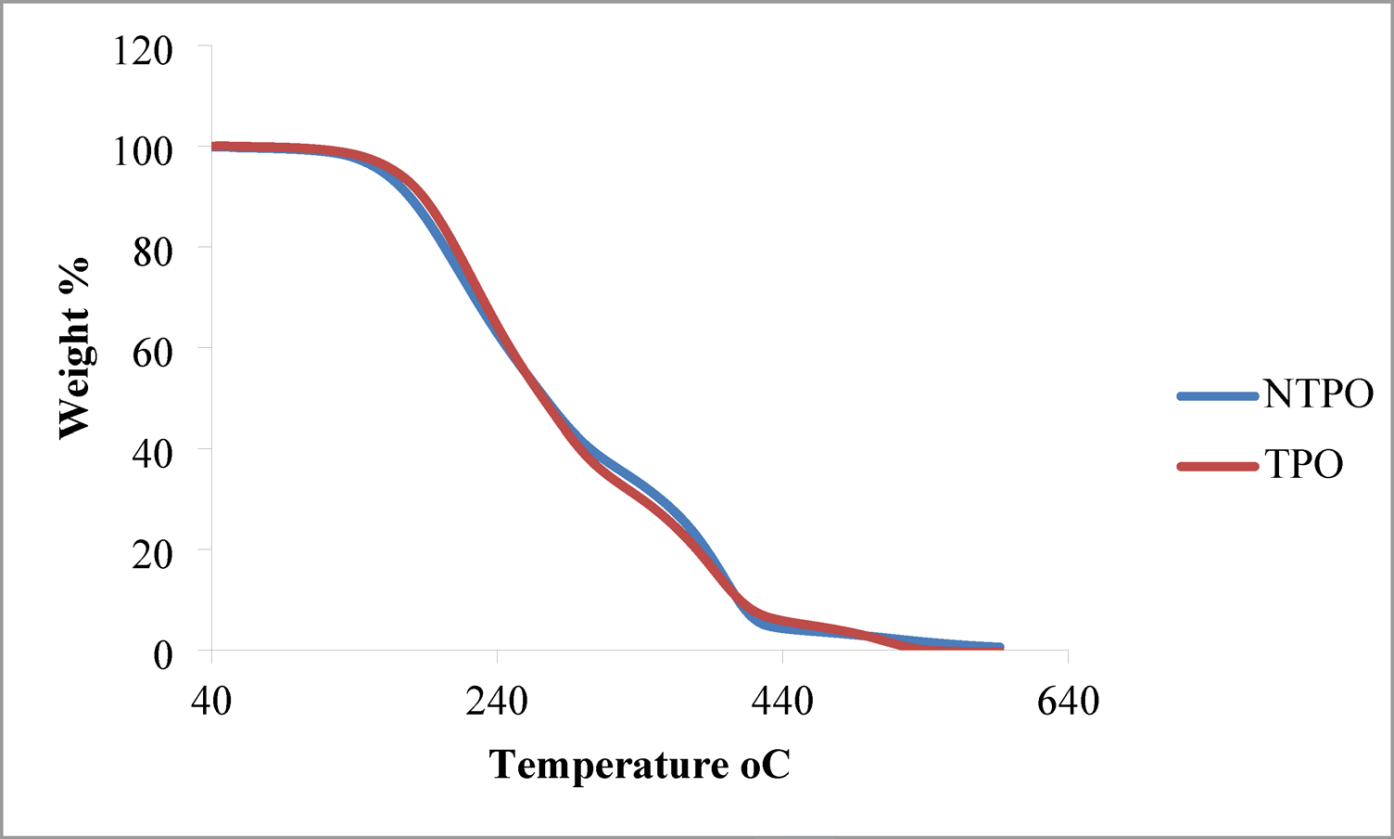
Thermal gravimetric analysis (TGA) curves of TPO and NTPO indicating their thermal stability profiles.

*TGA and DSC of TPO and NTPO* The thermal stability of the prepared terpolymer (TPO) and its nanocomposite (NTPO) was investigated by TGA, as presented in Fig. 5. Both materials exhibited a single-step major weight loss, indicating a relatively homogeneous degradation mechanism. For both TPO and NTPO, the initial thermal decomposition onset was observed around 240–260 °C, which corresponds to the breakdown of side chains and weak polymeric linkages. The major degradation stage occurred between 300 and 440 °C, representing backbone scission and volatilization of the organic matrix. Beyond 450 °C, only a minimal residue remained, confirming almost complete decomposition of the organic fraction. A comparison between the two curves shows that NTPO displays slightly improved thermal resistance in the early stages of degradation, with a slower weight-loss rate compared to pure TPO. This behavior can be attributed to the presence of nanobentonite, which provides a barrier effect, delaying thermal diffusion and enhancing polymer stability. Overall, both TPO and NTPO exhibit thermal stability up to 240 °C before degradation begins, which is significantly higher than the operating temperatures of diesel fuels. This confirms that the synthesized additives are stable under practical fuel application conditions and suitable for use as pour point depressants.

**Fig. 6:**
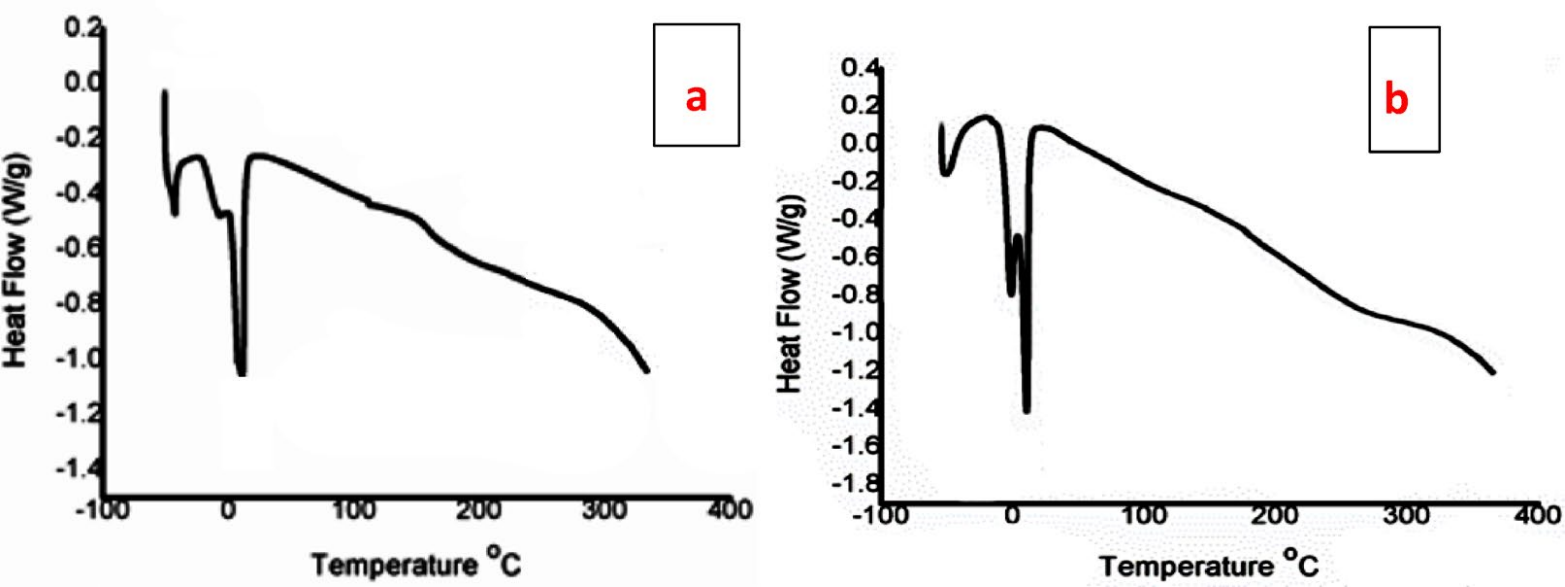
DSC thermograms of the (**a**) TPO and (**b**) NTPO.

The thermal behavior of the synthesized polymers was evaluated using DSC, as shown in Fig. 6a, b. For the TPO, a series of minor transitions were observed below 0 °C, which can be attributed to side-chain relaxations or partial crystallization of alkyl acrylate moieties. A distinct transition in the 0–20 °C range corresponds to the glass transition (T_g_) and possible melting of short-range ordered domains. Beyond this region, the curve displayed a gradual endothermic decline up to 300 °C, which is associated with the softening of the backbone and the onset of degradation. The absence of a sharp decomposition peak indicates good thermal stability. In contrast, the NTPO exhibited more intense exothermic events around 0 °C, suggesting a higher degree of side-chain crystallization and stronger ordering of the polymer segments. The deeper transitions also reflect a higher enthalpy change, consistent with increased crystallinity compared to the first polymer. Similar to the TPO, a broad endothermic trend extending from 100 to 300 °C was observed, indicating that both materials are thermally stable in this range. Comparatively, the TPO exhibits lower crystallinity and a more amorphous character, which can impart improved flexibility and low-temperature processability. The NTPO, with its higher crystallinity, may provide more structural rigidity but reduced flexibility at sub-ambient conditions. Nevertheless, both polymers demonstrate thermal stability with degradation onset above 250–300 °C, which is sufficient for their intended application as diesel fuel pour point depressant (PPD) additives.

### Impact of TPO and NTPO as PPDs

The pour point represents the minimum temperature at which diesel fuel retains its ability to flow under gravitational force. High pour points in waxy diesel fuels create challenges during cold-weather transportation due to paraffin wax crystallization. Pour point depressants work by altering wax crystal morphology, reducing their size and cohesion, which improves low-temperature fluidity^62–64^.

**Fig. 7:**
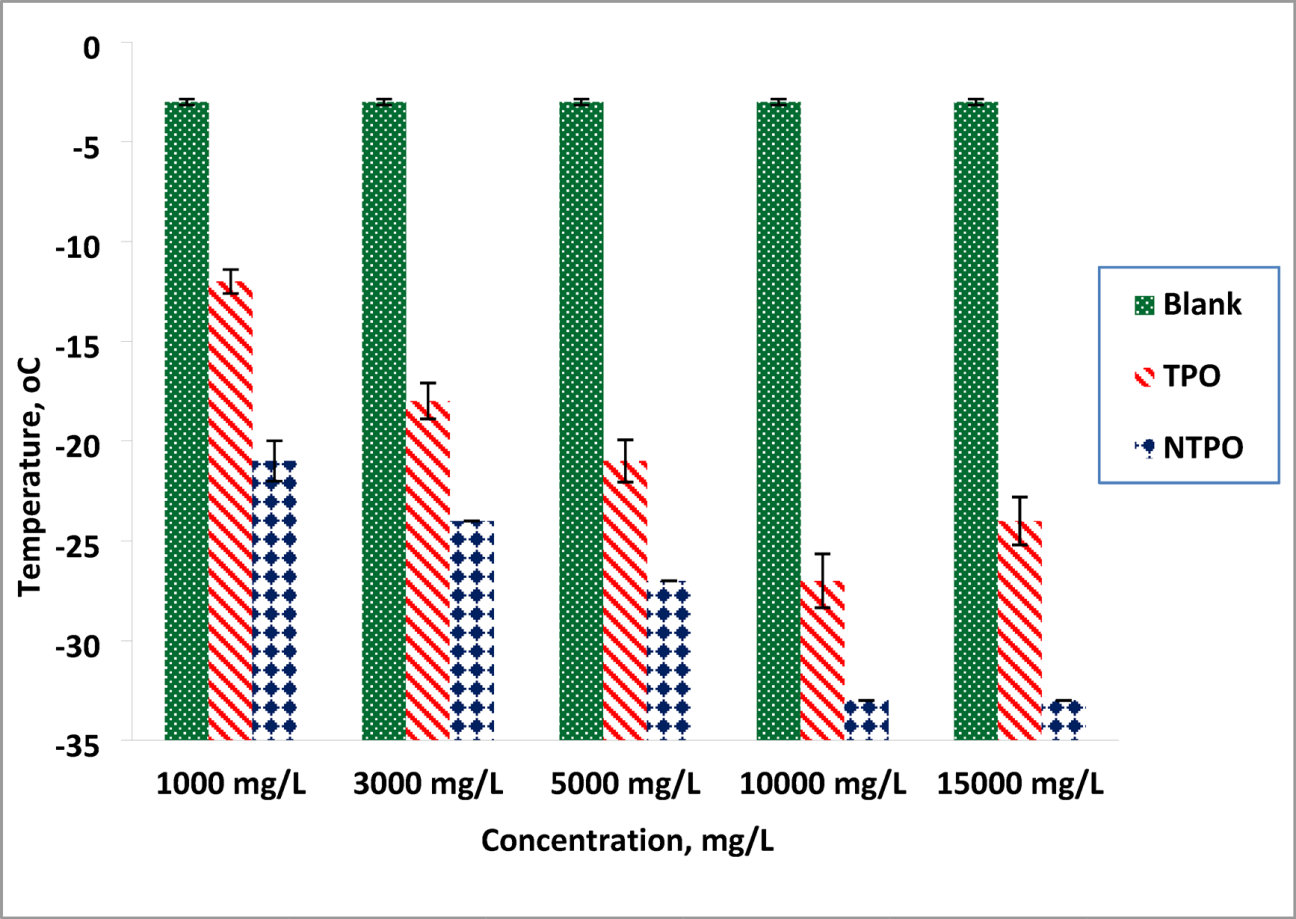
Variations in pour point temperatures of the treated and untreated diesel fuel.

The pour point temperatures of diesel fuel were measured after treatment with varying concentrations (1000, 3000, 5000, 10,000, and 15,000 mg/L) of TPO and NTPO additives, and measured according to ASTM D97 (measure the temperature every 3 °C until the oil stops flowing), then replicate the test 3 times to ensure the results. The experimental data, summarized in and illustrated in Fig. 7, revealed that NTPO exhibited greater effectiveness than TPO in reducing the pour point of diesel fuel. In case of treated diesel fuel, as the concentration of terpolymer increased, the treated diesel fuel oil’s pour point values gradually decreased. Therefore, it was found that utilizing 10,000 mg/L NTPO had the greatest potency for lowering the treated diesel fuel oil’s pour point from − 3 to – 33 °C. It was found that treatment with TPO resulted in a depression to − 27 °C. The higher concentration (15,000 mg/L) at TPO additive lead to slowly increase in the PP (− 24 °C) this may be attributed to the agglomeration of the polymer that hinder the dispersant of the wax but with using NTPO show the same results of 10,000 mg/L (− 33 °C) this is from to the effect of the nanobentonite that help the polymer to be more dispersed.

The enhanced performance at higher concentrations occurs through co-crystallization with paraffin wax, which modifies crystal morphology to prevent aggregation. At 10,000 mg/L, the additive effectively alters crystal structure, creating a barrier against three-dimensional wax network formation. In contrast, lower concentrations (< 10,000 mg/L) show reduced effectiveness because the terpolymer either: (1) acts as nucleation sites for wax crystallization, or (2) precipitates from solution^65^. This partial action at lower doses only slows lateral crystal growth without completely preventing wax formation^18^.

These results establish 10,000 mg/L NTPO as the most effective concentration for pour point depression, where complete crystal modification occurs to maximize low-temperature flow improvement in diesel fuels. NTPO’s superior efficiency stems from nanoparticle adsorption on wax crystals, which more effectively disrupts their 3D lattice formation. This nanoscale interaction enables fuel flow at significantly reduced temperatures, demonstrating the nanocomposite’s potential as a high-performance pour point depressant^66^.

*The cloud point (CP)* of the base diesel and samples treated with TPO and NTPO are shown in Table 5. The untreated diesel (blank) exhibited a CP of 6 °C. Addition of TPO reduced CP progressively to 3 °C at 10,000–15,000 mg L^−1^ (1.0–1.5 wt%), indicating improved resistance to wax appearance. The NTPO nanocomposite demonstrated superior performance: at the same dosages, CP fell to 2 °C, confirming that incorporation of nanobentonite enhances additive efficiency. These trends support the proposed mechanism whereby the terpolymer (long alkyl side chains) together with nanobentonite interferes with wax nucleation and growth, delaying the onset of visible crystallization (lower CP). The cloud point (CP) data show a clear reduction with increasing additive concentration for both TPO and NTPO, confirming their efficiency as pour point depressants. The low standard deviations (± 0.5 °C) across all measurements indicate high reproducibility and reliability of the experimental results, which enhances the credibility of the observed trends.

**Table 5:** Cloud point values of diesel fuel with and without TPO and NTPO additives ± SD and the molecular weight.

	Mwt g/mol	Blank	1000 mg/L	3000 mg/L	5000 mg/L	10,000 mg/L	15,000 mg/L
TPO	29,301	6 ± 0.5	5 ± 0.5	5 ± 0.5	4 ± 0.5	3 ± 0.5	3 ± 0.5
NTPO		6 ± 0.5	5 ± 0.5	4 ± 0.5	4 ± 0.5	2 ± 0.5	2 ± 0.5

**Fig. 8:**
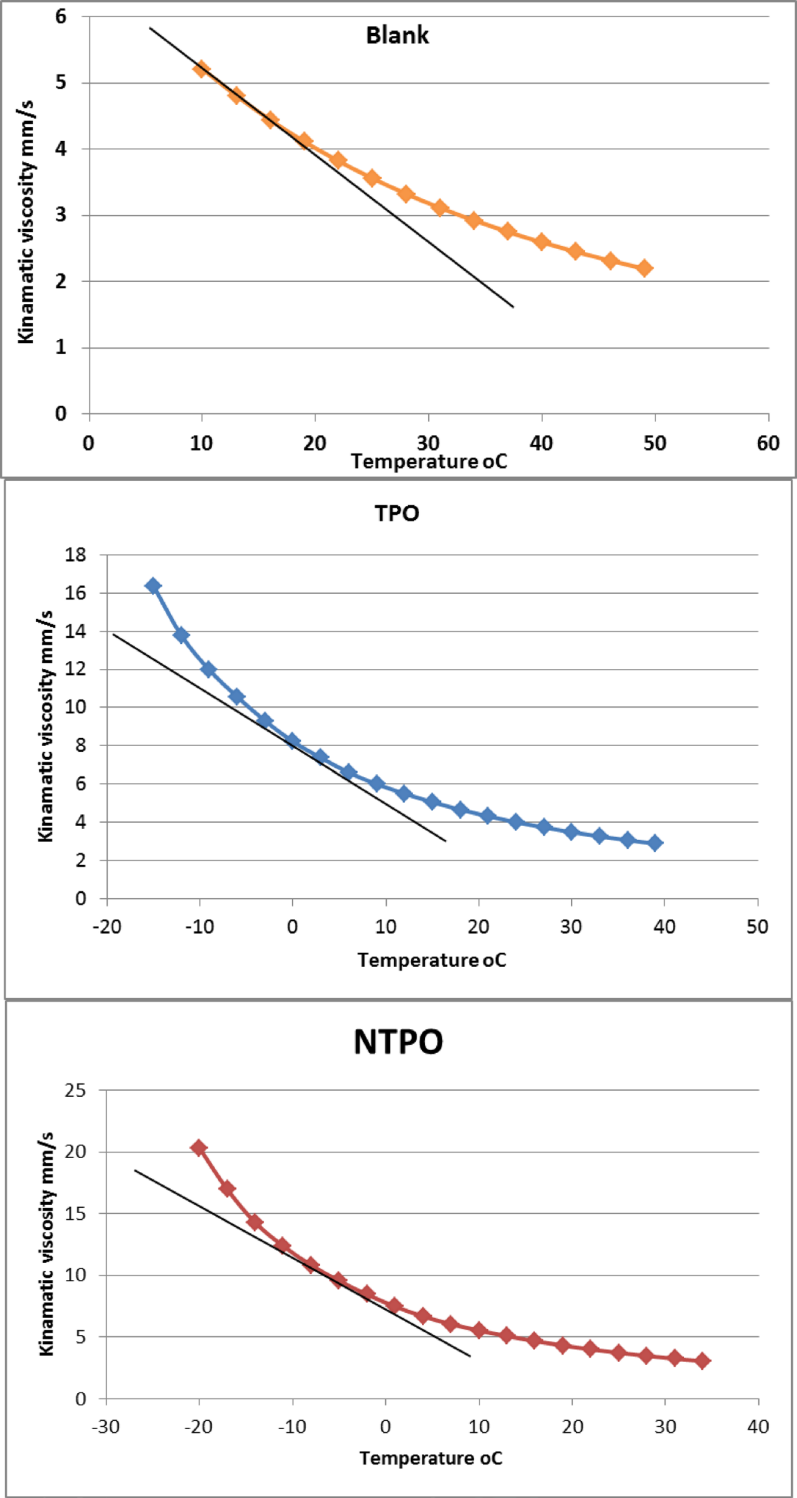
Viscosity–temperature curves of blank, TPO-treated, and NTPO-treated diesel samples.

*The wax appearance temperature (WAT)* The viscosity–temperature data were plotted, and the wax appearance temperature (WAT) was determined from the deviation point in the curves, as illustrated in Fig. 8. The wax appearance temperature (WAT) of the blank diesel sample was observed at 10 °C, indicating that wax crystals start to form at relatively high temperatures, which adversely affects the fuel flow properties under cold conditions. Upon the addition of TPO, the WAT significantly decreased to 3 °C, representing a reduction of about 7 °C compared to the blank. This pronounced shift confirms the efficiency of TPO in delaying the onset of wax crystallization by interfering with wax crystal nucleation and growth. Notably, the NTPO further reduced the WAT to − 5 °C, achieving an overall reduction of approximately 15 °C compared to the untreated fuel. This superior performance can be attributed to the synergistic effect of the nano-bentonite incorporated within the polymer matrix, which provides additional interaction sites with paraffin molecules and hinders crystal agglomeration. These findings clearly demonstrate that NTPO exhibits enhanced pour point depressant activity compared to TPO alone.

*The previous commercial studies* were compared with this study and tabulated in Table 6. The Table shows that conventional commercial PPDs such as EVA and PMA typically achieve 12–25 °C pour point reduction at dosages (0.1–0.5 wt%). In this study, the PP reduction achieved was 18–30 °C with the dose of the TPO/NTPO additives (0.1–1.0 wt%), the deduction at 0.1 wt% was 18 °C, and this is superior compared to the commercial to achieve this reduction at a relatively low concentration. Also, TPO/NTPO still depresses the temperature until it reaches the dose of 1 wt%. The achieved pour point depression was comparable to or better than some commercial benchmarks. This indicates potential practical utility, although optimization of synthesis and dosage is needed to reduce additive concentration and improve cost-effectiveness.

**Table 6:** Comparison between the pour point reduction of NTPO and other commercial compounds.

Additive	Typical dosage (wt%)	Pour point reduction (°C)	Reference
EVA (ethylene–vinyl acetate copolymer)	0.1–0.3	12–18	^73^
PMA (poly(meth)acrylate)	0.1–0.5	15–25	^12^
This work: NTPO	0.1–1	18–30	Present work

Although the additives demonstrated effective pour point depression in fresh diesel samples, future studies should investigate durability under real storage and thermal conditions, including sedimentation, storage stability, and thermal aging, to establish industrial feasibility.

### Evaluation of TPO and NTPO as viscosity index improvers (VII) in diesel fuel

The viscosity of diesel fuel is one of its most critical properties. As the temperature rises, the solvation power of diesel fuel increases, leading to a reduction in viscosity. This occurs because polymer molecules swell, increasing their hydrodynamic volume. Although this swelling compensates for the viscosity drop, the overall viscosity of the fuel still decreases^67^.

**Fig. 9:**
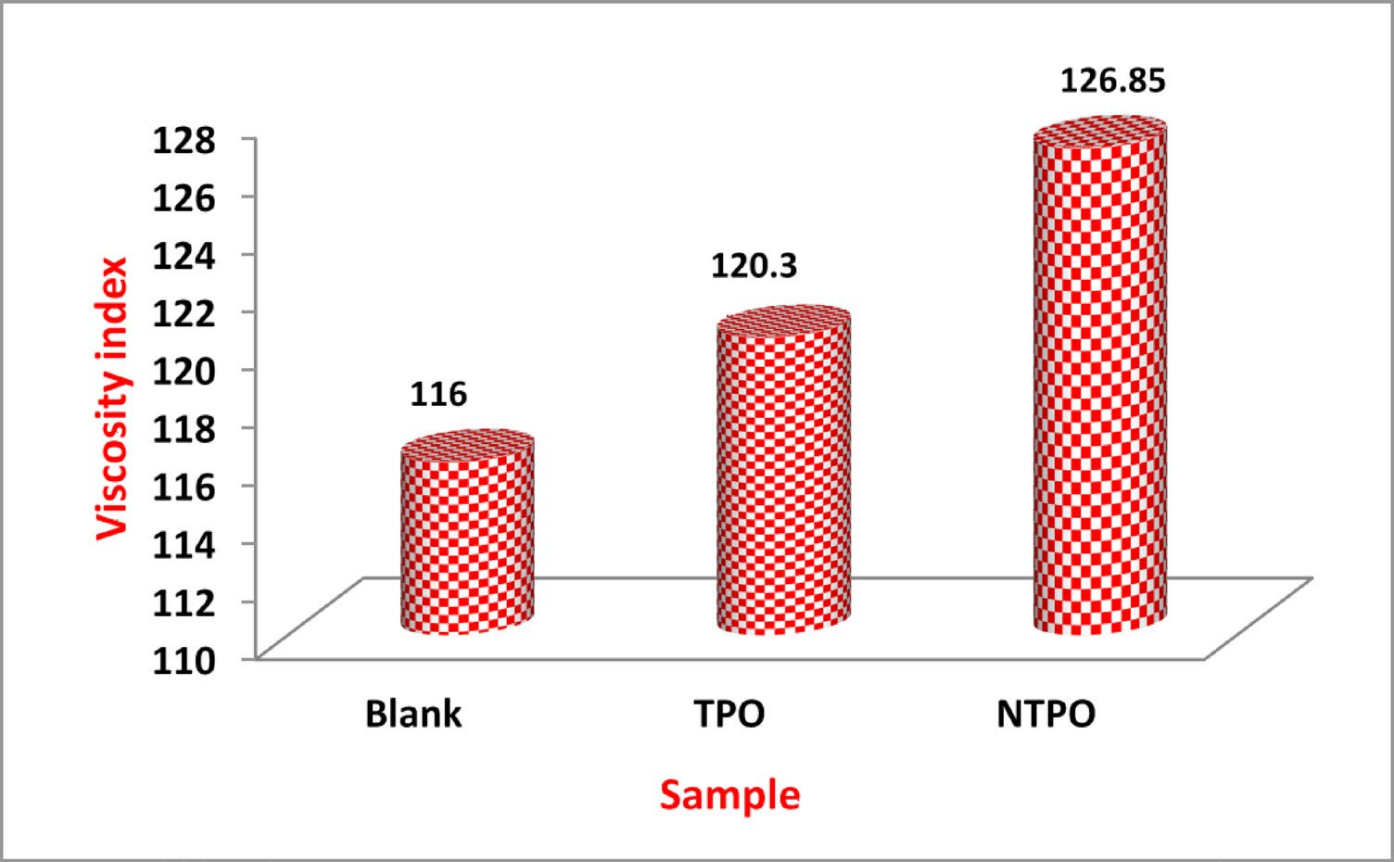
Variations in viscosity index of the treated and untreated diesel fuel.

Kinematic viscosity measurements were conducted on diesel fuel both with and without the addition of TPO and NTPO at a concentration of 10,000 mg/L, tested at 40 °C and 100 °C. The results, summarized in Table 7 and Fig. 9, demonstrate that both TPO and NTPO enhance the viscosity index (VI) of diesel fuel.

**Table 7:** The kinematic viscosity and viscosity index of the untreated diesel fuel and treated with prepared Terpolymer (TPO) and with polymer nano-bentonite (NTPO) at 10,000 ppm.

Sample	Kinematic viscosity, cSt ASTM D-445		Viscosity index
	40 °C	100 °C	
Blank	3.35	1.308	116
TPO	3.14	1.251	120.3
NTPO	3.06	1.231	126.85

When comparing the effectiveness of TPO and NTPO as viscosity index improvers (VII), the untreated diesel fuel had a VI of 116. However, treatment with either additive significantly improved the VI, with NTPO (126.85) outperforming TPO (120.3).

This study highlights the potential of TPO and NTPO as effective additives for improving the viscosity index of diesel fuel, with NTPO showing superior performance. Also, by comparison of this study with commercial diesel fuel as VI-improver, that summarized in Table 8, referring to the superiority of this work as VII.

**Table 8:** Comparison between the commercial Diesel fuel and the present work as VI-improver.

Fuel type	Kinematic viscosity @ 40 °C (cSt)	VI range	Reference standard
Diesel No. 1-D (Winter grade)	1.3–2.4	20–30	ASTM D975
Diesel No. 2-D (regular)	1.9–4.1	20–40	ASTM D975
EN 590 Diesel (Europe)	2.0–4.5	20–40	EN 590
Present study (NTPO)	3.06	126	ASTM D2270

### Rheological behavior and flow characteristics of diesel fuel oil

The rheological properties of diesel fuel exhibit shear-dependent behavior during cooling, where applied shear stress inhibits paraffin crystallization and maintains flow-ability. Below a critical shear rate threshold, progressive wax precipitation increases apparent viscosity and induces gel formation, potentially leading to pipeline flow cessation. When subjected to shear, interactions between polymeric molecules and wax crystals lead to crystal fragmentation, producing particles with varying sizes, shapes, and rheological properties. Therefore, addressing these flow challenges—particularly in cold environments—requires a thorough understanding of fuel oil rheology. The flow behavior is governed by compositional factors (wax type, quantity, and nature), crystallization behavior, and operational conditions, including applied shear rate, shearing duration, temperature, and cooling rate^67^.

**Fig. 10:**
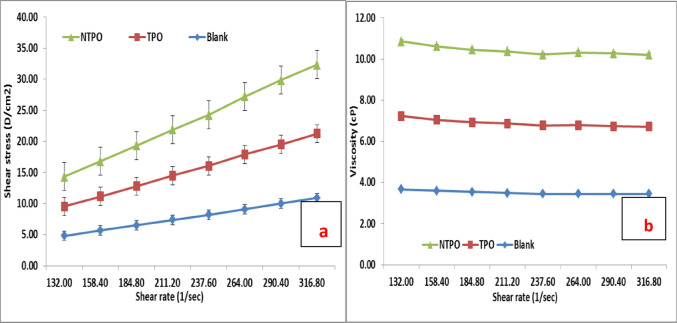
Shear rate versus (**a**) Shear stress and (**b**) Viscosity of treated and untreated diesel fuel at 20 °C.

**Fig. 11:**
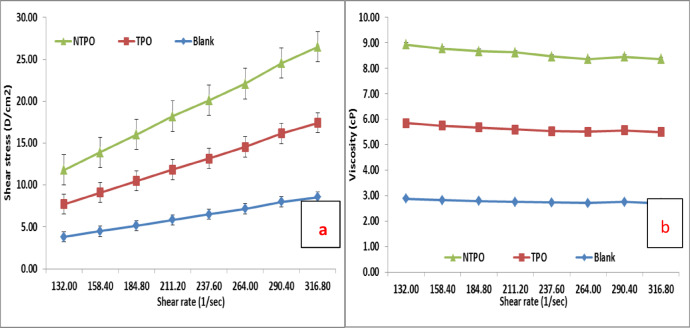
Shear rate versus (**a**) Shear stress and (**b**) Viscosity of treated and untreated diesel fuel at 30 °C.

**Fig. 12:**
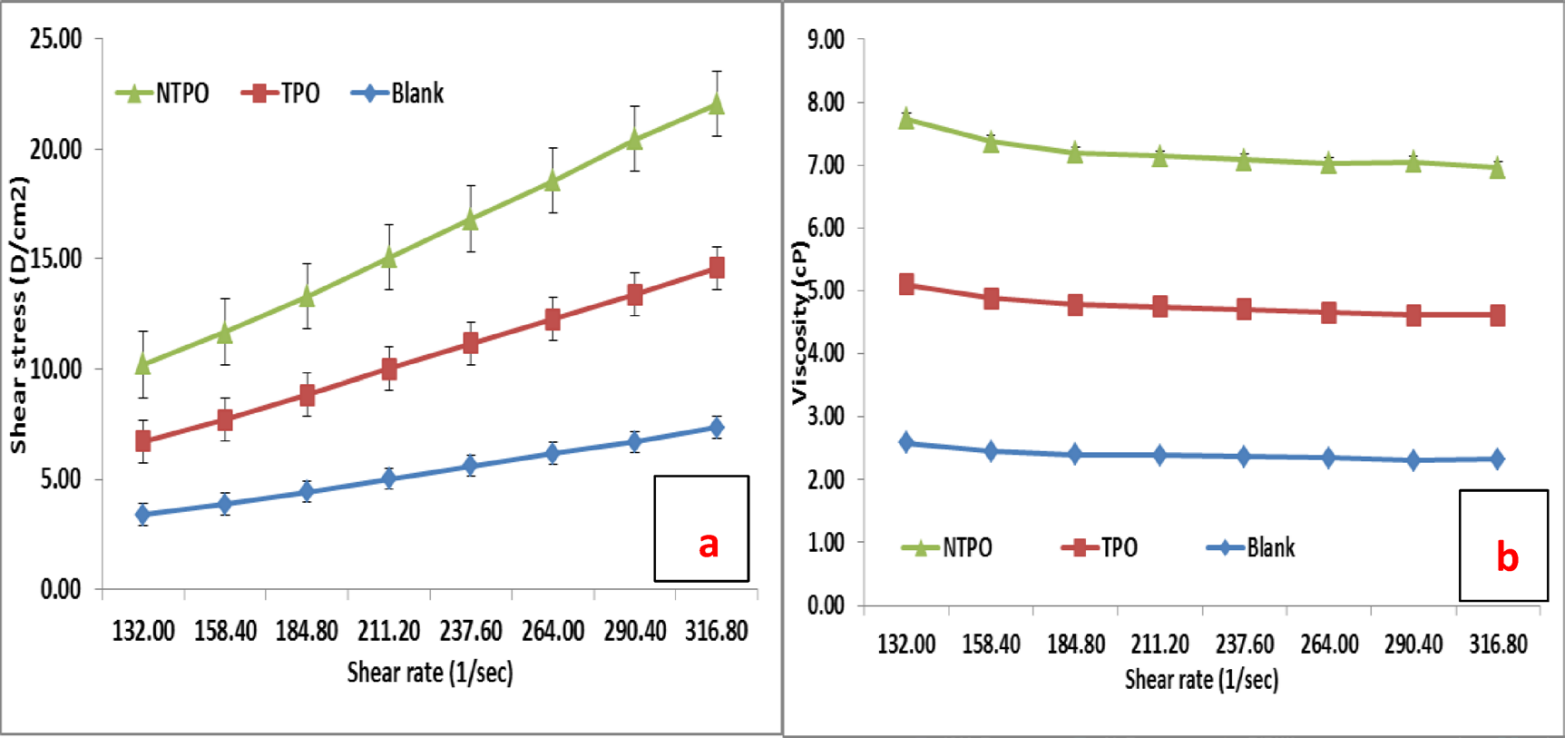
Shear rate versus (**a**) Shear stress and (**b**) Viscosity of treated and untreated diesel fuel at 40 °C.

This study conducted viscosity measurements for untreated and treated diesel fuel at 20 °C, 30 °C, and 40 °C, maintaining a consistent polymeric additive concentration of 10,000 mg/L. Figures 10, 11 and 12 illustrate variations in shear stress (Figs. 10, 11 and 12a) and viscosity (Figs. 10, 11 and 12a, b) as functions of shear rate. The experimental data demonstrate a proportional rise in shear stress as shear rate increases, consistent at all tested temperatures. The untreated fuel exhibits non-Newtonian, yield-pseudoplastic behavior, characterized by an abrupt rise in shear stress at higher shear rates. In contrast, treated diesel demonstrates a linear viscosity reduction with increasing shear rate, eventually stabilizing at elevated shear rates. Notably, NTPO outperforms TPO as a flow improver (FI), aligning with prior findings 68.

At temperatures near the pour point and under low shear, wax crystals undergo partial disruption due to applied shear energy. Higher shear rates enhance energy dissipation, overcoming yield stress and initiating flow. Agglomerated structures contract under intense shear, releasing trapped continuous phase components and reducing effective dispersed phase concentration, thereby lowering viscosity. This process continues until most agglomerates disintegrate to their primary size, explaining the non-Newtonian behavior of the oil systems^68,69^.

Elevating the temperature from 20 to 40 °C (20 °C, 30 °C, and 40 °C) reduces both shear stress and viscosity in treated diesel. Higher temperatures amplify interactions between polymeric additives and fuel, expanding the hydrodynamic volume of polymers and increasing their effective volume fraction. Consequently, viscosity diminishes. Additionally, thermal motion weakens paraffin particle networks, further contributing to reduced viscosity. These insights underscore the critical roles of shear history and temperature in optimizing diesel fuel flow properties.

Table 9 summarizes the rheological parameters of untreated diesel and diesel treated with TPO and NTPO additives at different temperatures (20, 30, and 40 °C). For the blank diesel sample, the apparent viscosity decreases consistently with increasing temperature (from 32.8 cP at 20 °C to 21.5 cP at 40 °C), which is the expected behavior for petroleum-based fuels. The yield stress values for blank diesel are low (< 1 Pa), indicating limited structural resistance to flow. In the case of TPO-modified diesel, the apparent viscosity values are slightly higher than the blank at low temperature, suggesting increased molecular interactions due to polymer incorporation. However, the yield stress values (1.64–1.54 Pa) are significantly higher than those of the blank, confirming that TPO enhances intermolecular structuring and delays flow initiation. For the NTPO nanocomposite system, the viscosity at 20–40 °C remains higher than that of untreated diesel, while the yield stress values are the highest among all samples (2.16–2.52 Pa). This indicates that the presence of nanobentonite provides additional sites for interaction with wax molecules, leading to stronger resistance against flow initiation. Notably, despite the increased yield stress, NTPO treatment effectively disrupts large wax crystal networks (as shown in microscopy and cold flow results), leading to improved low-temperature flowability.

**Table 9:** Rheological parameters (apparent viscosity in cP and yield stress in Pa) of blank, TPO-, and NTPO-treated diesel at different temperatures.

Sample	Temp. (°C)	Apparent viscosity (cP ) ± SD	Yield stress (Pa)	R^2^
Blank	20 30 40	32.8 ± 0.5 26 ± 0.4 21.5 ± 0.5	0.4666 0.3521 0.5095	0.99 0.99 0.98
TPO	20 30 40	30.6 ± 0.5 29.5 ± 0.45 21.1 ± 0.4	1.6368 0.1706 1.5373	0.99 0.98 0.97
NTPO	20 30 40	34.6 ± 0.5 32.5 ± 0.4 12.8 ± 0.4	2.1621 2.3449 2.5171	0.98 0.98 0.96

All rheological measurements were performed in triplicate, and the results showed high reproducibility with minor deviations, as reflected in the error bars and standard deviation in the Table. This confirms the reliability of the observed shear stress–shear rate and viscosity trends.

### Effect of TPO and NTPO on ash content

The ash content analysis provides important insight into the effect of additive incorporation on the combustion characteristics of diesel fuel. Table 10 summarizes the ash content of pure diesel fuel and that treated with TPO and NTPO. The blank diesel fuel exhibited negligible ash content (0.04%), confirming its clean-burning nature. After the addition of TPO, only a small change was detected (0.046%), which can be attributed to the organic polymeric structure of the additive. However, in the case of NTPO, the ash content increased slowly and noticeably (0.048%) due to the presence of nano-bentonite, an incombustible mineral filler. Although this increase remains within acceptable fuel specification limits, it reflects the partial contribution of inorganic residues after combustion. This outcome emphasizes the importance of balancing the advantages of wax crystal growth inhibition with the potential side effect of higher ash content in nanocomposite-based additives.

**Table 10:** Ash content in the diesel fuel and that treated with 10,000 mg/L of TPO and NTPO.

Sample	Ash content %
Blank	0.04
TPO	0.046
NTPO	0.048

### Effect of additive composition on wax crystal morphology in diesel fuel

Microscopic image analysis corroborates conventional flow property assessments by examining wax crystallization patterns in untreated and treated diesel fuel. This technique was employed to evaluate the performance of synthesized bifunctional polymeric additives (designed to function as combined pour point depressants and wax inhibitors) based on their impact on wax crystallization behavior.

Pour point depressants work by adsorption on the surface of wax crystals. The resulting surface layer of the pour point depressants prevents the development of wax crystals and their ability to absorb oil and form gels^17^. Photomicrographic analysis affirms other standard flow experiments, which assess the pour point depressants of treated/untreated diesel oil through the crystallization behavior of wax.

**Fig. 13:**
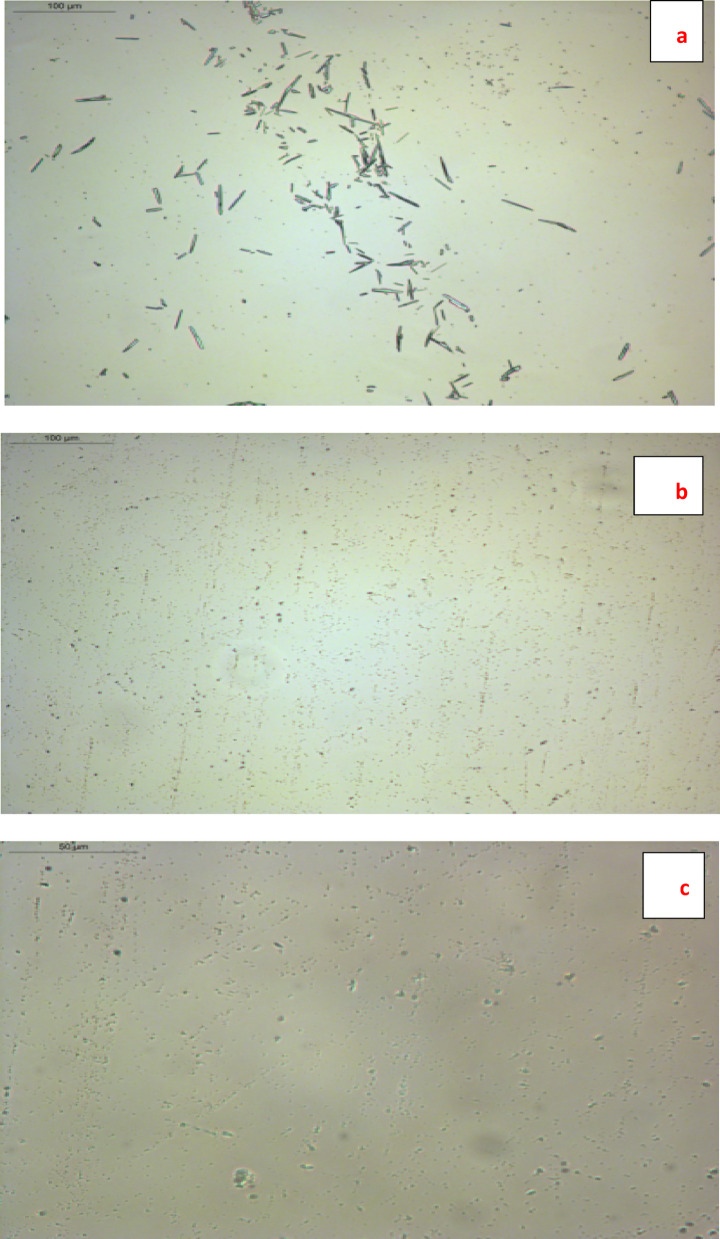
Polarized optical microscopy image (POM) of the wax of diesel fuel samples with Magnification: 200 × (**a**) blank with scale bar = 100 µm, (**b**) TPO with scale bar = 100 µm and (**c**) NTPO with scale bar = 50 µm.

Figure 13a–c presents comparative photomicrographs of untreated diesel fuel versus samples treated with TPO and NTPO additives. Untreated fuel (Fig. 13a) exhibited large, well-defined paraffin crystal aggregates with an average size of 25.5 ± 2.7 µm, indicating uncontrolled wax precipitation. In contrast, treated samples (Fig. 13b, c) displayed significantly smaller and more uniformly dispersed crystalline particles, demonstrating enhanced paraffin dispersion. Upon addition of TPO, the wax crystals became significantly smaller and more dispersed, with an average size of 3.08 ± 0.30 µm. For the NTPO sample, the wax crystals were further reduced to nanoscale dimensions (0.79 ± 0.13 µm), indicating strong inhibition of wax growth. The substantial reduction in crystal size reflects the efficiency of the prepared additives in modifying crystal morphology and suppressing aggregation, thereby enhancing the cold flow properties of diesel fuel.

The microscopic evidence reveals that both TPO and NTPO additives effectively modify crystallization behavior by creating multiple nucleation sites. This multi-point attachment mechanism disrupts paraffin crystal development, resulting in finer particulate distribution and consequent reduction in the fuel’s pour point. The improved dispersion characteristics confirm these additives’ efficacy in controlling wax-related flow problems in diesel fuels.

### Proposed mechanism of wax deposition prevention

**Fig. 14:**
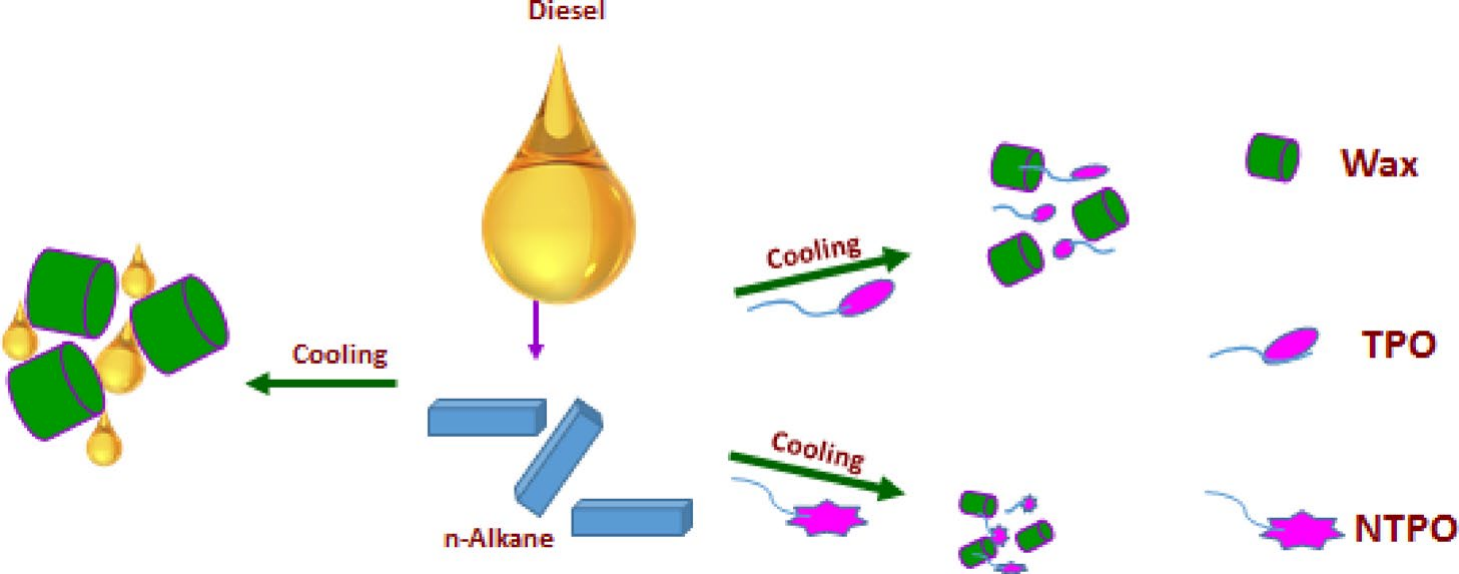
An inferred mechanism for TPO and NTPO improving the cold flow properties of diesel fuel.

Figure 14 presents the proposed mechanism for how TPO and NTPO additives enhance diesel fuel’s cold flow properties. In untreated diesel, decreasing temperatures cause n-alkanes to precipitate as wax crystals. These crystals develop into large, disk-shaped structures that interconnect to form extensive three-dimensional networks. This crystalline framework traps liquid fuel molecules, significantly reducing fluidity. The additives (TPO and NTPO) modify this process by co-crystallizing with wax crystals, altering their crystallization behavior and disrupting their normal growth patterns.

The chemical additives play a crucial role in inhibiting wax crystal growth and promoting the formation of smaller crystals with high surface-to-volume ratios. A Terpolymer that contains long chains present in different three monomers used; hexadecyl acrylate, octolyate and α-olefins adsorbed on the wax molecules (paraffin) and act as the first nuclei instead of the nucleation of the wax itself. The nuclei formed repulsed from the other free wax molecule through Van der Waals forces with the aid of the polar groups in the monomers. The repulsion of wax molecules and the nuclei delays and alters the lattice formation. The delay in the formation of the crystal lattice is highly pronounced in the terpolymer nanocomposite because of the inorganic nanobentonite that helps in the formation of another Site for repulsion. Wax crystallization is typically inhibited or delayed through various mechanisms involving nucleation interference, co-crystallization, or adsorption of these additives onto nascent crystals, preventing their aggregation^70^. An alternative theory suggests that surface modification phenomena and thermodynamic solubility work synergistically to enhance flow properties in diesel fuel treated with polymeric additives^71,72^. In the present work, we assume that the prepared additives adsorb onto wax crystals via Van der Waals forces and organize themselves to restrict crystal growth through steric hindrance, as illustrated in Fig. 14.

The mechanism of cold-flow improvement can be directly correlated with the shifts in WAT, CP, and PPT. For the untreated diesel, wax crystals start to appear at relatively high temperatures (WAT = 10 °C, CP = 6 °C), leading to poor flowability at sub-ambient conditions (PPT =  − 3 °C). The addition of TPO significantly delayed the onset of wax crystallization (WAT = 3 °C, CP = 3 °C, PPT =  − 27 °C), indicating that the polymer molecules interact with paraffin chains and interfere with crystal nucleation and growth. A further improvement was observed with NTPO (WAT =  − 5 °C, CP = 2 °C, PPT =  − 33 °C), which confirms the synergistic role of nano-bentonite. The nanoparticles likely provide additional heterogeneous sites that disturb the regular packing of paraffin molecules, restrict crystal size, and prevent large-scale agglomeration. This dual effect of polymer adsorption and nanoparticle disruption explains the superior cold-flow performance of NTPO compared to TPO alone.

## Conclusion

This research applied the newly synthesized terpolymer TPO and its nanobentonite NTPO as highly efficient pour point depressants and flow enhancers for diesel fuels. These polymers were synthesized, and their structures were proved through many analyses: IR, ^1^H NMR, GPC, DLS, TEM, and EDX. Thermal degradation was studied, and the stability of the two polymers, especially the NTPO, by integrating these functionalized polymers with modified nano-bentonite with oleic acid particles, the additives effectively suppress wax crystallization and modify crystal structure, significantly improving low-temperature flow properties. They depress the pour point from − 3 to – 27 °C and – 33 °C with the optimum concentration (10,000 mg/L) by using TPO and NTPO, respectively. Viscosity–temperature analysis confirmed that the additives effectively lowered the wax appearance temperature (WAT) of diesel, shifting it from 10 °C (blank) to 3 °C (TPO) and − 5 °C (NTPO). These results demonstrate that NTPO provides superior inhibition of wax crystallization, which is in agreement with its enhanced cold-flow performance. The viscosity index increased from 116 to 120 and 126 for both polymers. Most of the studies refer to the superiority of NTPO over TPO. The synergistic interaction between polymer chains and well-dispersed nanoparticles reduces the pour point and optimizes viscosity behavior at sub-zero temperatures. Compared to conventional PPDs, the developed additives exhibited superior performance, offering a more efficient and cost-effective solution for cold-weather diesel applications. These findings suggest that further optimization of polymer structure and nanoparticle loading could lead to commercially viable additives tailored for specific fuel formulations and climate conditions. Although this work focused on one diesel sample, our future studies on diverse diesel grades (e.g., low-sulfur, high-paraffin, biodiesel blends) will help extend the applicability of these findings, and also will investigate the durability under real storage and thermal conditions; sedimentation, storage stability, and thermal aging, to establish industrial feasibility.

## Supplementary Information

Below is the link to the electronic supplementary material.

Supplementary Material 1

## Data Availability

The datasets used and/or analyzed during the current study are available from the corresponding author, Abeer A. El-Segaey.

## References

[1] Vellaiyan, S. Optimization of spirulina biodiesel-ammonium hydroxide blends with exhaust gas recirculation for enhanced diesel engine performance and emission reduction. *Results Eng.* **26**, 105015 (2025).

[2] Xu, B. et al. Terpolymers of alkyl methacrylate-trans anethole-1,2,3,6-tetrahydrophthalic anhydride copolymers: A low dosage and high-efficiency cold flow improver for diesel fuel. *Chin. Chem. Lett.* **36**, 110196 (2025).

[3] Menezes, E., da Silva, R., Cataluña, R. & Ortega, R. Effect of ethers and ether/ethanol additives on the physicochemical properties of diesel fuel and on engine tests. *Fuel* **85**, 815–822 (2006).

[4] Shi, E. et al. Research status and challenges of mechanism, characterization, performance evaluation, and type of nano-pour point depressants in waxy crude oil. *ACS Omega* **9**, 35256–35274 (2024). 39184475 10.1021/acsomega.4c05243 PMC11339835

[5] Aljohani, B. S., Aljohani, K., Kandasamy, M., Vellaiyan, S. & Nagappan, B. Enhancement of diesel engine performance and emission reduction using ZnS nanoparticles and water emulsions with electrostatic precipitator integration. *Case Stud. Therm. Eng.* **71**, 106157 (2025).

[6] El-Segaey, A. A., El-Azabawy, R. E., Mohammed, H. A., Al-Shafey, H. I. & Kamal, R. S. Comparative study between a copolymer based on oleic acid and its nanohybrid for improving the cold flow properties of diesel fuel. *ACS Omega* **8**, 10426–10438 (2023). 36969437 10.1021/acsomega.2c08294 PMC10035025

[7] Yang, T. et al. Influence of polar groups on the depressive effects of polymethacrylate polymers as cold flow improvers for diesel fuel. *Fuel* **290**, 120035 (2021).

[8] Nadirov, K. et al. Novel pour point depressants for crude oil derived from polyethylene solution in hexane and coal fly ash. *Fluids* **9**, 121 (2024).

[9] El-Shamy, A.E.-H.A., Khidr, T. T. & Badr, E. E. Studies the effect of hydrazide derivatives as flow improvers for waxy crude oil. *Pet. Coal* **61**, 533–539 (2019).

[10] Khidr, T. T. Alternating copolymerized derivative as pour point depressant for gas oil. *Pet. Coal* **62**, 1420–1426 (2020).

[11] Yang, T. et al. Effects of N-containing pour point depressants on the cold flow properties of diesel fuel. *Fuel* **272**, 117666 (2020).

[12] Chen, J., Cui, L., Xu, B., Lin, H. & Han, S. Influence of polymers with surfactant properties as pour point depressants on the cold flow properties of diesel fuel. *Colloids Surf. A Physicochem. Eng. Asp.* **677**, 132390 (2023).

[13] Wang, H. et al. Evaluated the effects of nature α-olefins (limonene, β-caryophyllene and camphene) as additives on the cold flow properties of diesel fuel. *J. Mol. Liq.* **409**, 125486 (2024).

[14] Guin, S. & Naiya, T. K. Enhancement of cold flowability of waxy crude oil using eco-friendly PPDs synthesized from stearic acid and lauric acid—Experimental, modelling, and mechanistic approach. *J. Mol. Liq.* **426**, 127363 (2025).

[15] Elkatory, M. R. et al. Influence of poly (benzyl oleate-co-maleic anhydride) pour point depressant with di-stearyl amine on waxy crude oil. *Polymers* **15**, 306 (2023). 36679187 10.3390/polym15020306 PMC9865987

[16] Patel, N. C., Valodkar, V. B., Pantar, A. V. & Tembe, G. L. A review of nanocomposite based polymeric pour point depressants for crude flow assurance. *Polym. Technol. Mater.* **64**, 1506–1537 (2025).

[17] Al-Sabagh, A., Sabaa, M., Saad, G., Khidr, T. T. & Khalil, T. M. Synthesis of polymeric additives based on itaconic acid and their evaluation as pour point depressants for lube oil in relation to rheological flow properties. *Egypt. J. Pet.* **21**, 19–30 (2012).

[18] Al-Sabagh, A. M., Khidr, T. T., Moustafa, H. M., Mishrif, M. R. & Al-Damasy, M. H. Investigating the synergistic effect between oil soluble surfactants and styrene–maleic anhydride copolymers to enhance the flow properties of waxy crude oil. *Pet. Sci. Technol.* **35**, 1381–1388 (2017).

[19] Modi, P. & Nagar, A. Synthesis of terpolymer from Helianthus annuus (natural oil) and evaluation as pour point depressant and viscosity reducer on Indian crude oil. *J. Dispers. Sci. Technol.* **45**, 1039–1048 (2024).

[20] Ahmed, S. M., Khidr, T. T. & Ismail, D. A. Effect of gemini surfactant additives on pour point depressant of crude oil. *J. Dispers. Sci. Technol.* **39**, 1160–1164 (2018).

[21] Khidr, T. T. & Omar, A. M. A. Anionic/nonionic mixture of surfactants for pour point depression of gas oil. *Egypt. J. Pet.* **13**, 21–26 (2003).

[22] Xie, Y. et al. Performance and mechanism of a novel electro-magnetic treatment for improving the cold flowability of waxy crude oil. *Fuel* **382**, 133803 (2025).

[23] Wang, H. et al. Effects of comb-like poly-α-olefins on the cold flow properties of diesel fuel. *Fuel* **356**, 129562 (2024).

[24] Xie, M. et al. Synthesis and evaluation of benzyl methacrylate-methacrylate copolymers as pour point depressant in diesel fuel. *Fuel* **255**, 115880 (2019).

[25] Yuan, D. et al. Synthesis and performance testing of maleic anhydride-ene monomers multicomponent co-polymers as pour point depressant for crude oil. *Polymers* **15**, 3898 (2023). 37835947 10.3390/polym15193898 PMC10574900

[26] Nikolaev, A. & Plotnikova, K. Study of the rheological properties and flow process of high-viscosity oil using depressant additives. *Energies* **16**, 6296 (2023).

[27] Elganidi, I., Elarbe, B., Abdullah, N. & Ridzuan, N. Synthesis of a novel terpolymer of (BA-co-SMA-co-MA) as pour point depressants to improve the flowability of the Malaysian crude oil. *Mater. Today Proc.* **42**, 28–32 (2021).

[28] Sun, B., Chen, F., Lin, H., Xue, Y. & Han, S. Influence of comb type terpolymers of methyl benzyl acrylate-co-hexadecene-maleic anhydride with tetradecyl pendant on cold flow properties of diesel fuel. *Colloids Surf. A Physicochem. Eng. Asp.* **658**, 130636 (2023).

[29] Al-Sabagh, A. M., El-Hamouly, S. H., Khidr, T. T., El-Ghazawy, R. A. & Higazy, S. A. Synthesis of phthalimide and succinimide copolymers and their evaluation as flow improvers for an Egyptian waxy crude oil. *Egypt. J. Pet.* **22**, 381–393 (2013).

[30] Song, Y., Ren, T., Fu, X. & Xu, X. Study on the relationship between the structure and activities of alkyl methacrylate–maleic anhydride polymers as cold flow improvers in diesel fuels. *Fuel Process. Technol.* **86**, 641–650 (2005).

[31] Kang, J. et al. Cold flowability improvement of waxy crude oil doped with graphene nanoparticles and its mechanism. *J. Mol. Liq.* **405**, 125083 (2024).

[32] Khidr, T. T., Keshawy, M. & Abdel-Raouf, M. Pour point depressants for waxy crude oil based on used sun flower oil. *Egypt. J. Chem.* **67**, 83–94 (2024).

[33] Kurniawan, M., Norrman, J. & Paso, K. Pour point depressant efficacy as a function of paraffin chain-length. *J. Pet. Sci. Eng.* **212**, 110250 (2022).

[34] Sun, B. et al. Synthesis of methacrylate–vinyl acetate–N-phenylmethylpropionamide terpolymers as pour point depressants and combined with methyl palmitoleate to improve the cold flowability of waste cooking oil biodiesel blends. *J. Mol. Liq.* **368**, 120796 (2022).

[35] Xue, Y., Yang, T., Lin, H., Zheng, S. & Han, S. Effect of methacrylate-methacrylamide copolymers with various polar pendants on the cold flow properties of diesel fuels. *Fuel* **315**, 123112 (2022).

[36] Zhang, X. et al. Synthesis of nano-hybrid polymethacrylate-carbon dots as pour point depressant and combined with ethylene-vinyl acetate resin to improve the cold flow properties of diesel fuels. *Energy* **253**, 124186 (2022).

[37] Yin, S. et al. Influence of tetradecyl methacrylate-n-α-methacrylamide copolymers as pour point depressants on the cold flow property of diesel fuel. *Energy Fuels* **34**, 11976–11986 (2020).

[38] Yang, F. et al. Performance improvement of the ethylene-vinyl acetate copolymer (EVA) pour point depressant by small dosages of the polymethylsilsesquioxane (PMSQ) microsphere: An experimental study. *Fuel* **207**, 204–213 (2017).

[39] Alves, B. F., Rossi, T. M., Marques, L. C. C., Soares, B. G. & Lucas, E. F. Composites of EVA and hydrophobically modified PAMAM dendrimer: Effect of composition on crystallization and flow assurance of waxy systems. *Fuel* **332**, 125962 (2023).

[40] Han, S., Wang, P., Wang, Y., Song, Y. & Ren, T. Impact of alkyl methacrylate–maleic anhydride–alkyl methacrylate terpolymers as cold flow improver on crystallization behavior of diesel fuel. *Process Saf. Environ. Prot.* **88**, 41–46 (2010).

[41] Chen, F. et al. Influence of maleic anhydride-co-methyl benzyl acrylate copolymers modified with long-chain fatty amine and long-chain fatty alcohol on the cold flow properties of diesel fuel. *Fuel* **268**, 117392 (2020).

[42] Faujdar, E., Negi, H., Singh, R. K. & Varshney, V. K. Study on biodegradable poly(α-olefins–co–α-pinene) architectures as pour point depressant and viscosity index improver additive for lubricating oils. *J. Polym. Environ.* **28**, 3019–3027 (2020).

[43] Feng, L., Zhang, Z., Wang, F., Wang, T. & Yang, S. Synthesis and evaluation of alkyl acrylate-vinyl acetate-maleic anhydride terpolymers as cold flow improvers for diesel fuel. *Fuel Process. Technol.* **118**, 42–48 (2014).

[44] El-Segaey, A. A., Arafa, E., El-Tabey, A. E., Hashem, A. & Al-Shafey, H. Influence of the terpolymer and its nanocomposite pour point depressants on lubricating oil properties. *Pet. Petrochem. Eng. J.* **3**, 1–12 (2019).

[45] Al-Shafey, H. I. et al. Comparative strategy between masterly flow improver and its nanocomposite. *Polym. Bull.* **79**, 2725–2745 (2022).

[46] El-Bahnasawi, A. H. et al. Comparison between methacrylate copolymers and their magnetite nanocomposite as pour point depressant for lubricating base oil. *Discov. Appl. Sci.* **6**, 69 (2024).

[47] Han, X., Li, T., Liu, G. & Vellaiyan, S. Comparative evaluation and economic analysis of metal- and carbon-based nanoadditives in low-viscous waste-derived biofuel blends for diesel engines. *Case Stud. Therm. Eng.* **69**, 106057 (2025).

[48] Yao, B. et al. Organically modified nano-clay facilitates pour point depressing activity of polyoctadecylacrylate. *Fuel* **166**, 96–105 (2016).

[49] Sharma, R., Mahto, V. & Vuthaluru, H. Synthesis of PMMA/modified graphene oxide nanocomposite pour point depressant and its effect on the flow properties of Indian waxy crude oil. *Fuel* **235**, 1245–1259 (2019).

[50] Jia, X. et al. Submicron carbon-based hybrid nano-pour-point depressant with outstanding pour point depressant and excellent viscosity depressant. *Arab. J. Chem.* **15**, 104157 (2022).

[51] Yu, H. et al. Effect of a magnetic nanocomposite pour point depressant on the structural properties of daqing waxy crude oil. *Energy Fuels* **33**, 6069–6075 (2019).

[52] Xue, Y. et al. Effect of nanocomposite as pour point depressant on the cold flow properties and crystallization behavior of diesel fuel. *Chin. Chem. Lett.* **33**, 2677–2680 (2022).

[53] Vellaiyan, S. Comparative evaluation of nanoparticle-enriched Gossypium hirsutum methyl ester blends for enhanced energy, emission, and economic performance in diesel engines. *Clean. Eng. Technol.* **27**, 101004 (2025).

[54] El-Bahnasawi, A. H. et al. Evaluation of newly copolymers and their montmorillonite nanocomposite as cold flow improver for petroleum lubricating oil. *Sci. Rep.* **13**, 14991 (2023). 37696841 10.1038/s41598-023-41802-1 PMC10495333

[55] Betiha, M. A., Mahmoud, T. & Al-Sabagh, A. M. Effects of 4-vinylbenzyl trioctylphosphonium- bentonite containing poly(octadecylacrylate-co-1-vinyldodecanoate) pour point depressants on the cold flow characteristics of waxy crude oil. *Fuel* **282**, 118817 (2020).

[56] Patel, Z., Patel, J. & Nagar, A. Investigation of styrene based terpolymers as pour point depressant for waxy crude oils. *Egypt. J. Pet.* **33**, 3 (2024).

[57] Elbanna, S. A., Abd El Rhman, A. M., Al-Hussaini, A. S. & Khalil, S. A. Paraffin inhibition and pour point depression of waxy crude oil using newly synthesized terpolymeric additives. *Egypt. J. Chem.* **65**(131), 1455–1463 (2022).

[58] Patel, Z., Patel, J. & Nagar, A. Role of acrylate terpolymers in flow assurance studies of crude oil. *Pet. Sci. Technol.* **43**, 1597–1619 (2025).

[59] Samsalykovich, K. S., Abdirasululy, Z. A., Kainarbaevich, D. B., Ruslanovna, M. A. & Bakytuly, A. T. Synthesis and modification of pour point depressant (PPD) based on copolymers of α-olefins and maleic anhydride for waxy crude Oil BT—Proceedings of the 6th International Conference on Fundamental and Applied Sciences. in (eds. Abdul Karim, S. A., Abd Shukur, M. F., Fai Kait, C., Soleimani, H. & Sakidin, H.) 27–35 (Springer Nature Singapore, Singapore, 2021).

[60] Al-Shafy, H. Studies on the influence of polymeric additives as flow improvers for waxy crude oil. *IOSR J. Eng.* **4**, 54–61 (2014).

[61] Mahmoud, T. & Betiha, M. A. Poly(octadecyl acrylate-co-vinyl neodecanoate)/oleic acid-modified nano-graphene oxide as a pour point depressant and an enhancer of waxy oil transportation. *Energy Fuels* **35**, 6101–6112 (2021).

[62] Tang, X. et al. Internal causes and characteristics of high-pour-point oil non-Darcy percolation in porous media under cold damage: An analytical and experimental study. *Fuel* **379**, 133001 (2025).

[63] Quan, Q., Jin, W., Sun, N., Wang, Y. & Wang, S. Effect of ethylene vinyl acetate on pour point and wax deposition characteristics of high-pour-point and viscous waxy crude oil. *Fuel* **395**, 135269 (2025).

[64] Li, H. & Zhang, J. A generalized model for predicting non-Newtonian viscosity of waxy crudes as a function of temperature and precipitated wax☆. *Fuel* **82**, 1387–1397 (2003).

[65] Khidr, T. T., Azzam, E. M. S., Mutawaa, S. S. & Omar, A. M. A. Study of some anionic surfactants as pour point depressants additives for a waxy gas oil. *Ind. Lubr. Tribol.* **59**(2), 64–68 (2007).

[66] Meneghetti, P. & Qutubuddin, S. Synthesis, thermal properties and applications of polymer-clay nanocomposites. *Thermochim. Acta* **442**, 74–77 (2006).

[67] Mehrotra, A. K., Ehsani, S., Haj-Shafiei, S. & Kasumu, A. S. A review of heat-transfer mechanism for solid deposition from “waxy” or paraffinic mixtures. *Can. J. Chem. Eng.* **98**, 2463–2488 (2020).

[68] Taheri-Shakib, J., Shekarifard, A. & Naderi, H. The experimental investigation of effect of microwave and ultrasonic waves on the key characteristics of heavy crude oil. *J. Anal. Appl. Pyrolysis* **128**, 92–101 (2017).

[69] Franco, C. A. et al. Enhancing heavy crude oil mobility at reservoir conditions by nanofluid injection in wells with previous steam stimulation cycles: Experimental evaluation and field trial implementation. *J. Mol. Liq.* **424**, 127024 (2025).

[70] Wei, B. Recent advances on mitigating wax problem using polymeric wax crystal modifier. *J. Pet. Explor. Prod. Technol.* **5**, 391–401 (2015).

[71] El-Tabey, A. E., El-Segaey, A. A., Khidr, T. T. & Elsharaky, E. A. Sustainable conversion of polystyrene waste into polymeric surfactants to be evaluated as pour point deperssants for waxy crude oil. *J. Mol. Liq.* **411**, 125780 (2024).

[72] Paso, K., Krückert, K., Oschmann, H., Ali, H. & Sjöblom, J. PPD architecture development via polymer-crystal interaction assessment. *J. Pet. Sci. Eng.* **115**, 38 (2014).

[73] Liu, J. et al. Effect of poly(ethylene-vinyl acetate) pour point depressant on the cold flow properties and crystallization behavior of soybean biodiesel blends fuel. *Turk. J. Chem.* **46**, 311–319 (2022). 38143472 10.3906/kim-2106-49 PMC10734696

