# Supplementary material for: Synergistic effects of terpolymer and its oleic modified nano-bentonite nanocomposite for cold flow enhancement for diesel fuel
Source: Sci Rep. 2025 Nov 10;15:39369. doi: 10.1038/s41598-025-24090-9 (PMC12603330; doi:10.1038/s41598-025-24090-9)
Supplement: Supplementary file 1 — Supplementary Material 1 [file 41598_2025_24090_MOESM1_ESM.docx]

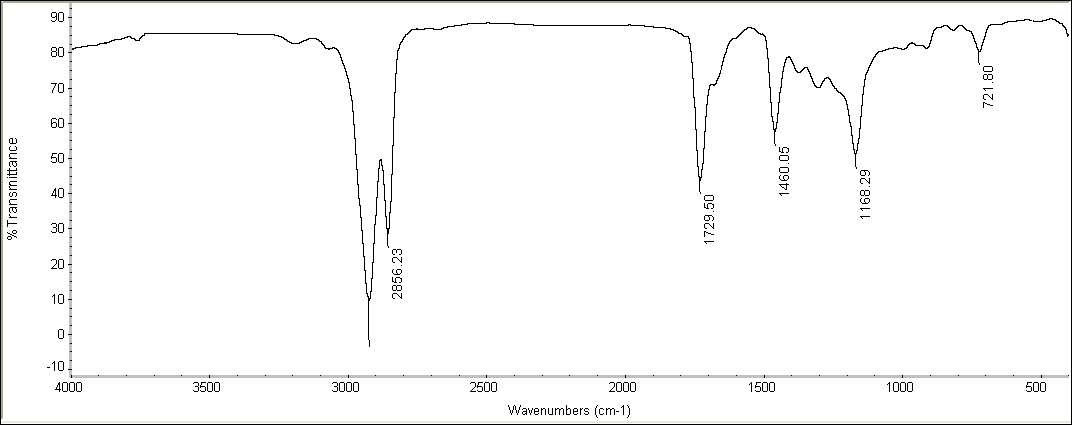


Figure (1): FT-IR of TPO


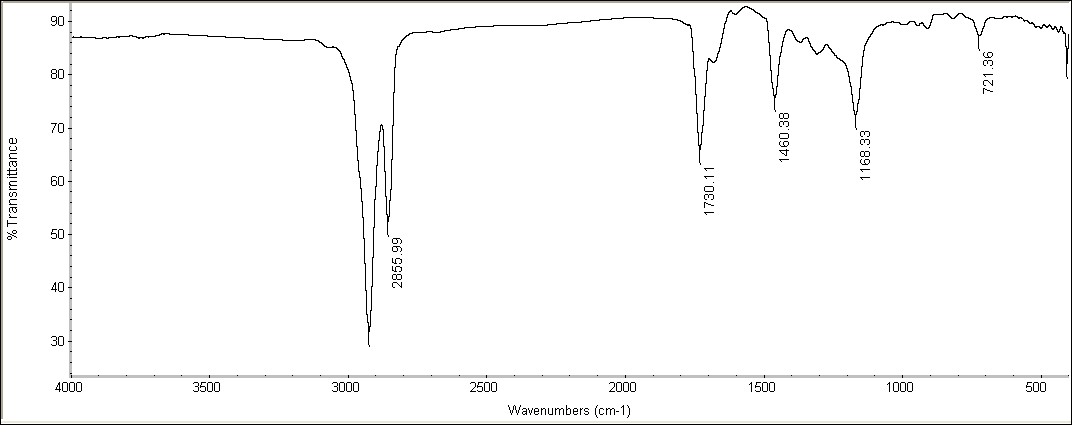


Figure (2): FT-IR of NTPO


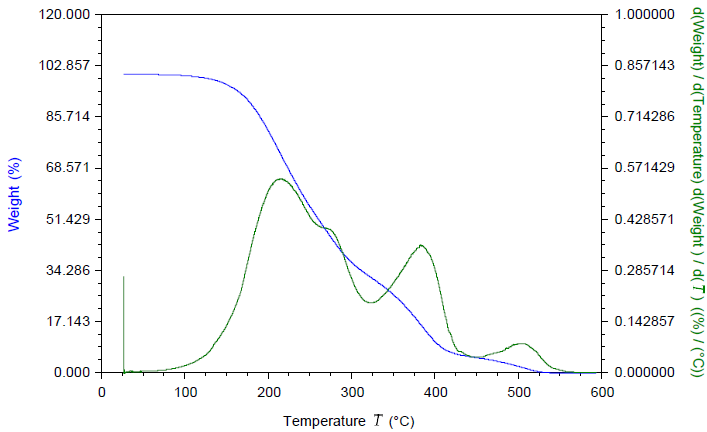


Figure (3): TGA of TPO


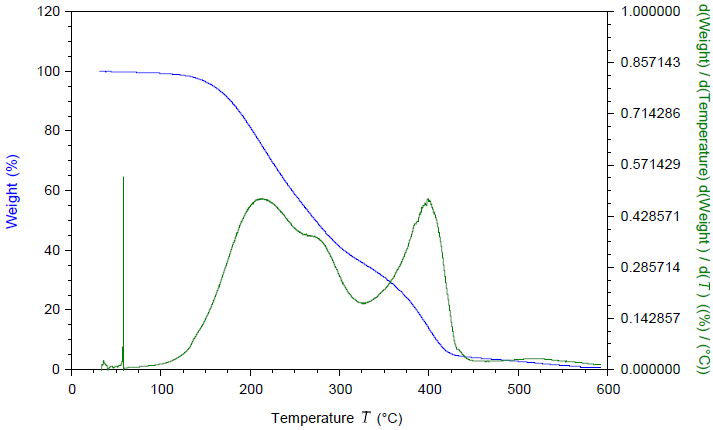


**Figure (4): TGA of NTPO**

**Table (1)**: Pour point temperatures (PPT)±SD of untreated diesel fuel (-3 ^o^C) and treated with different concentrations of the additives

|  | 1000mg/L | 3000mg/L | 5000mg/L | 10000mg/L | 15000mg/L |
| --- | --- | --- | --- | --- | --- |
| TPO | -12±3 | -18±3 | -21±3 | -27±3 | -24±3 |
| NTPO | -21±3 | -24±3 | -27±3 | -33±3 | -33±3 |
